# Strong links promote the emergence of cooperative elites

**DOI:** 10.1038/s41598-019-47278-2

**Published:** 2019-07-26

**Authors:** Edoardo Gallo, Yohanes E. Riyanto, Tat-How Teh, Nilanjan Roy

**Affiliations:** 1 Faculty of Economics, University of Cambridge, Sidgwick Avenue, Cambridge, CB3 9DD UK; 2 Queens’ College, CB3 9ET Cambridge, UK; 3 Division of Economics, School of Social Sciences, Nanyang Technological University, 48 Nanyang Avenue, HSS #04-70, Singapore, 639818 Singapore; 4 Department of Economics, Faculty of Arts and Social Sciences, National University of Singapore, AS2 06-02 1 Arts Link, Singapore, 117570 Singapore; 5 Department of Economics and Finance, College of Business, City University of Hong Kong, 83 Tat Chee Avenue, Kowloon Tong, Hong Kong

**Keywords:** Mathematics and computing, Human behaviour

## Abstract

The maintenance of cooperative behavior is fundamental for the prosperity of human societies. Empirical studies show that high cooperation is frequently associated with the presence of strong social ties, but they are silent on whether a causal mechanism exists, how it operates, and what features of the social environment are conducive to its emergence. Here we show experimentally that strong ties increase cooperation and welfare by enabling the emergence of a close-knit and strongly bound cooperative elite. Crucially, this cooperative elite is more prevalent in social environments characterized by a large payoff difference between weak and strong ties, and no gradation in the process of strengthening a tie. These features allow cooperative individuals to adopt an all or nothing strategy to tie strengthening based on the well-known mechanism of direct reciprocity: participants become very selective by forming strong ties only with other cooperative individuals and severing ties with everyone else. Once formed, these strong ties are persistent and enhance cooperation. A dichotomous society emerges with cooperators prospering in a close-knit, strongly bound elite, and defectors earning low payoffs in a weakly connected periphery. Methodologically, our set-up provides a framework to investigate the role of the strength of ties in an experimental setting.

## Introduction

Cooperation among individuals is a fundamental driver of human prosperity^1,2^, and there are longstanding theoretical and experimental literatures investigating the factors driving its emergence using variants of a prisoner’s dilemma^3^. Many contexts displaying a high level of cooperation in the real world are characterized by the presence of strong ties based on family^4,5^, ethnicity^6,7^, or social relations^4,8^. Well-known examples from empirical studies include commerce in medieval^6^ and modern^9^ times, diamond trading^4^, criminal organizations^7^, and hunter-gatherer societies^8,10^. This frequent coexistence of strong ties and high levels of cooperation raises the question of whether the ability to form strong ties is a causal determinant of cooperative behavior.

The bulk of theoretical studies on the co-evolution of network structure and cooperation has abstracted away from the strength of ties and focused on unweighted networks^11^. These studies show how the availability of information about others’ reputation and the ability to rapidly rewire the network are important determinants of cooperation in unweighted networks where a link is either present or absent^12–14^. There are, however, a few simulation-based studies that extend these models to settings with weighted ties where the weight represents the frequency of interactions between agents or a multiplicative factor applied to the payoffs^15–18^. Although the simulations differ in the details of their set-up and assumptions, they all show that the introduction of weighted ties increases the level of cooperation. In particular, they show that this increase in cooperation occurs because cooperators tend to cluster together and they are connected by strong ties leading to a positive correlation between cooperative behavior and having strong ties.

These theoretical predictions are consistent with the empirical evidence that strong social ties are present when cooperation emerges, but no study to date has investigated the existence of a causal channel. Experimental studies of the causal determinants of cooperative behavior have so far focused on other factors which are often coexisting with strong ties such as reputational systems^19–24^, simple binary ties^25–31^, knowledge of the social network^30^, or the timing of decisions^32^. At the moment we ignore whether strong ties play a causal role in promoting cooperative behavior, what features of strong ties are important, and what are the underlying mechanisms of tie formation leading to close-knit, strongly connected groups of cooperators.

The gap in our understanding of the causal role of the strength of social ties extends beyond the domain of cooperation. In recent years, a growing number of experimental studies have investigated the causal impact of network structure on individual behavior, but all of these studies focus on unweighted networks where a tie is either present or absent^33^. A potential reason for this gap is that it is not clear how one can represent strong ties in a laboratory setting. Our study proposes a novel methodology to investigate experimentally whether strong ties promote cooperative behavior in a prisoner’s dilemma (PD) game played on a dynamic network. The strength of a social tie is represented by a scaling factor applied to the payoffs of the game so if the tie between two individuals becomes twice as strong then they play a PD game whose payoffs are multiplied by a factor of two. A direct interpretation is that it captures the essence of a strong tie as an interaction entailing higher stakes both in the gains and losses domains. An alternative interpretation is that the doubling of the strength of a tie means the individuals interact twice as much so the number of times they play the game is equal to the scaling factor. These two interpretations are consistent with the theoretical models and theoretically equivalent as long as the payoffs of the PD game are symmetric around 0 because the scaling factor does not affect the relative incentives between gains and losses. From an experimental point of view, however, these two interpretations may not be behaviorally equivalent and therefore we adopt the former direct interpretation hereafter. In general, this experimental methodology can be used to explore the role of strong ties in other games with analogous payoff symmetry, and it may be extended to other types of games if one is careful about dealing with the non-linearities involved in scaling the payoffs.

There are 4 treatments that explore two separate dimensions of a social environment: the strength of the tie and the number of gradations of strengths available. In the baseline there is no possibility to strengthen ties so participants can only connect through weak ties. In the medium tie treatment participants have the option to double the strength of an existing tie, while in the strong tie treatment participants can quadruple the strength of an existing tie. Both these treatments represent social environments where there are only two gradations of strength so a tie is either weak or medium/strong. In a final medium-strong tie treatment, a weak tie can be strengthened to a medium tie and then a medium tie can be strengthened to a strong one so there are three gradations of strength. These variations allow us to shed light on the efficacy of tie strength in promoting cooperation in different environments, and they facilitate the identification of the dynamics of link formation and strengthening that lead to this causal relation. At aggregate level, the main outcomes of interest are the level of cooperation, total payoffs and the structure of the network. At the individual level we are interested in understanding the strategies individuals use to form/cut connections, and the impact they have on the emerging network structure.

## Experiment Setup

**Figure 1:**
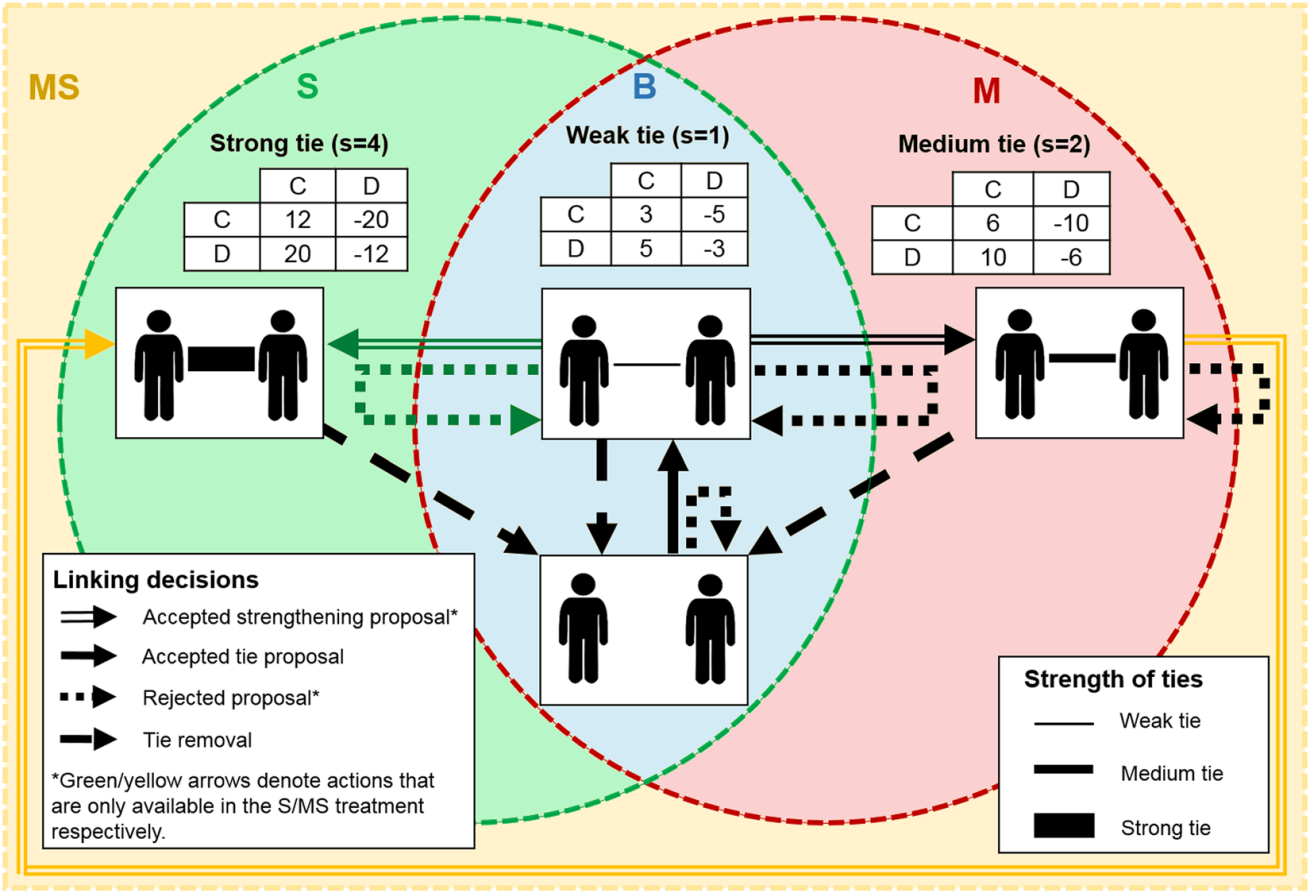
The experiment consists of 4 treatments and the options available to participants in each treatment are delineated by sets of different colors. In the (blue) baseline *B*, participants start without any ties (bottom pair) and have the option to form a tie to play the game with *s* = 1. In addition to the options available in *B*, in the (red) Medium *M* treatment participants can strengthen a tie to play the game with *s* = 2. The (green) Strong *S* treatment is analogous to *M*, but the strengthening of a tie means playing a game with *s* = 4. Finally, the (yellow) Medium and Strong *MS* treatment encompasses all the options available in the other treatments: participants can strengthen a tie to play the *s* = 2 game and then strengthen it further to play the *s* = 4 game. Notice that the option to strengthen from *s* = 1 directly to *s* = 4 is only available in the *S* treatment.

We recruited 384 students to participate in a laboratory experiment. The set-up of the experiment is a modification of the one used in Gallo and Yan (2015)^30^. Each participant is assigned to a group of 12 and plays at least 25 rounds of a PD game on a network that is formed by the participants themselves. The first round starts with the empty network. After round 25, there is a 50% chance that the game terminates in each of the following rounds. Each round consists of three stages. The first two stages determine the network on which participants play the game, while in the third stage participants play a PD game with their neighbours in the network. Figure 1 is a schema of the experimental design with the 4 treatments denoted by different colours. All the treatments are between-subjects so each subject participates in one and only one treatment.

The Baseline (*B*) treatment is represented in the blue set of Fig. 1. In stage 1, participants can propose costless ties to any of the other participants and can unilaterally remove any of their existing ties. There is no limit to the number of ties each participant can remove or propose. When two participants both propose a tie to each other then the tie is added, which is indicated by a solid black arrow pointing from the unconnected to the connected box. Conversely, a participant removing a tie is indicated by a dashed black arrow pointing from the connected to the unconnected box. If a participant has proposed a tie to another participant who has not done the same, then in stage 2 the recipient of the proposal can accept or reject the proposal. An accepted proposal leads to the formation of a tie and therefore it is denoted by a solid black arrow, while a rejected one is denoted by a dotted black line pointing back to the unconnected box. At the end of stage 2, the network is updated with all the linking decisions. In stage 3 participants play the PD game in the blue subset of Fig. 1, which is completely symmetric in payoffs: (*C*, *C*) gives 3 points to each participant, (*D*, *D*) gives −3 points, (*C*, *D*) gives 5 points to the defector and −5 points to the cooperator, and both participants get 0 points if they are not linked. They play the PD game against other individuals with whom they have formed a tie. Specifically, they play a bilateral PD game with each of these linked neighbors. Participants do not choose separate actions each round for each partner they play with, but instead they choose an action to implement against all of their neighbors. Participants are also informed of the last three decisions of other players who are linked to them, which they may use to end and/or strengthen the existing ties^31^.

In addition to the choices available in *B*, the other treatments allow participants to strengthen their ties. The strength of a tie is represented by a scaling factor applied to the payoffs of the game: if two participants have a tie of strength *s* then they play a game in which (*C*, *C*) gives 3*s* points to each participant, (*D*, *D*) gives −3*s* points, (*C*, *D*) gives 5*s* points to the defector and −5*s* points to the cooperator, and both participants get 0 points if they are not linked. In the Medium (*M*) and Strong (*S*) treatments, participants can form weak ties as well as ties with two (*s* = 2) and four (*s* = 4) times the strength of weak ties respectively. The red and green sets in Fig. 1 delineate the choices available in *M* and *S* respectively, which include all the choices available in *B*. The decision to strengthen a tie is similar to the decision to form a tie. After 3 consecutive rounds in which they have been connected by a weak tie, in stage 1 each participant can costlessly propose to strengthen the tie. The 3 rounds requirement to strengthen a tie represents the fact that typically this process takes time in the real world. In stage 2, the other participant can accept or reject the strengthening proposal. An accepted proposal is indicated by a hollow arrow pointing from the weak tie to the medium/strong tie box, while a rejected proposal is denoted by a dotted line pointing back to the weak tie box. The removal of a medium or strong tie is the same as the removal of a weak tie: it is unilateral and it disconnects the two participants, as shown by the dashed black line pointing to the unconnected box. Finally, in the Medium and Strong (*MS*) treatment in the yellow set in Fig. 1 participants can be connected by weak (*s* = 1), medium (*s* = 2) and strong (*s* = 4) ties: they have to be connected by a weak tie for 3 consecutive rounds to strengthen it to a medium tie, and by a medium tie for 3 consecutive rounds to strengthen it to a strong tie.

At the end of a round participants receive the following information: a reminder of the action they chose, the actions chosen by each neighbor, and the points they receive from the game with each of the other participants. The current network is carried over to the next round. At the end of the experiment for each participant we select 12 pairings between the participants and one of the other participants (2 random pairings in each of 6 randomly selected rounds) and convert the sum of points won in those pairings into dollars for payment. This random selection of pairs for payment, independently of whether a connection exists or not, ensures that there are uniform incentives throughout the experiment in forming connections so the payment system does not introduce biases in the emerging network structure. For instance, if we had excluded unconnected pairs from the random selection for payment, then participants would have incentives to form just one tie with a cooperator to ensure that a specific pairing is picked. This choice is in contrast to studies that pay the cumulative number of points participants have earned^26,28,29^, which may lead to satisficing in the latest rounds and therefore lower incentives to change the network.

Aside from its suitability to the application of our novel methodology to represent tie strength in an experimental setting, the selection of a payoff structure symmetric around 0 provides the correct incentives to allow the formation of meaningful and realistic network structures because the symmetry of payoffs in the gains/losses domains means that both the absence of a connection and connections between a defector and a cooperator lead to no change in social surplus, i.e. the sum of payoffs of all the participants. The only way to produce social surplus is a connection between two cooperators, and, conversely, the only way to reduce social surplus by an equal amount is a connection between two defectors. This is in contrast to studies that have non-negative^29,31^ or small negative^26,28^ payoffs, which lead to the emergence of overconnected networks because the losses from being connected to a defector are non-existent or negligible.

## Results

The starting point of the analysis is a comparison of differences in cooperation level, payoffs and network structure across treatments. We use the Kruskal-Wallis test to investigate the presence of treatment effects, and then supplement it with the Dunn’s test to check for differences between treatment pairs (see *SI*, Table S4). Notice that the aggregation at the group level leaves us with *n* = 8 independent observations per treatment so the detection of a statistically significant effect is indicative of a large treatment effect. For each comparison we report *P* values for both the Kruskal-Wallis (*KW*) and Dunn’s (*DT*) tests. The *SI* contains all the details and P values as well as a check that the results are robust to applying a Benjamini-Hochberg correction for multiple comparisons. The aggregate analysis focuses on rounds 8–23: after round 8 the network stabilizes in all treatments (see *SI*, section 3) and we exclude the latest rounds to avoid end-game effects.

**Figure 2:**
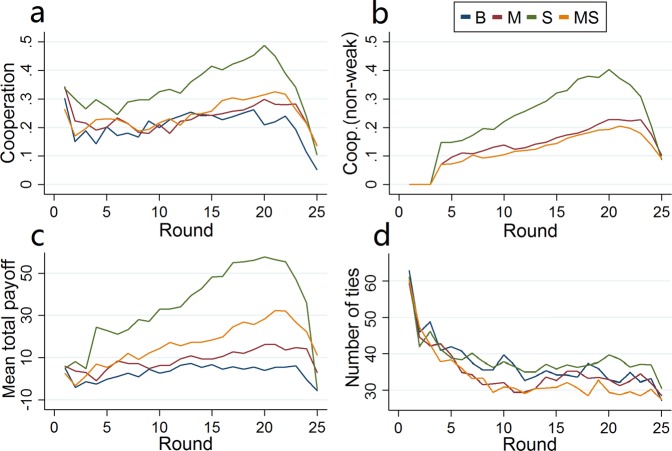
Cooperation level for (**a**) all ties and (**b**) non-weak ties, (**c**) mean total payoff, and (**d**) number of ties over 25 rounds for treatments *B* (blue), *M* (red), *S* (green) and *MS* (yellow).

Figure 2a plots the dynamics of the level of cooperation, and it shows that the possibility to form strong ties without any gradation in the *S* treatment is crucial to achieve a high level of cooperation. While an initial Kruskal-Wallis test does not directly confirm any significant treatment differences (*KW*, *P* = 0.286), pairwise comparisons show that participants in the *S* treatment, where only weak and strong ties are available, achieve a significantly higher level of cooperative activity than the *B* (*DT*, *P* = 0.05) and *M* (DT, *P* = 0.04) treatments, and qualitatively higher than the *MS* condition. There is no significant difference in the cooperation level in any comparison among the *B*, *M* and *MS* treatments. The differences in cooperation level between *S* and the other treatments are mostly driven by the strong links. Figure 2b shows the dynamics of the level of cooperation taking into account only the non-weak links, and therefore excluding the baseline *B*. The level of cooperation of participants in the *S* treatment is higher than in the *M* (KW, *P* = 0.088; DT, *P* = 0.06) and *MS* (DT, *P* = 0.03) treatments, and there is no significant difference between *M* and *MS*.

The consequence of the differences in cooperative activity is that participants in the *S* treatment achieve a higher level of welfare, defined as the sum of the participants’ payoffs (KW, *P* = 0.007). Figure 2c plots the evolution of the mean total payoff, and it shows that in *S* it is twice as large as in *MS* (DT, P = 0.06), three times larger than in *M* (DT, *P* = 0.005) and four to five times larger than *B* (DT, *P* = 0.001). There is no significant difference in welfare between any other treatment pair, except that it is marginally higher in *MS* compared to *B* (DT, *P* = 0.04).

Despite the difference in cooperation and welfare between *S* and the other treatments, the features of the networks that emerge are very similar at the aggregate level. Figure 2d shows the evolution of the number of ties in the network. There is no significant difference at the 5% level between any two treatments for this metric. There are also minimal differences across a range of network metrics including the clustering coefficient, the number of components, the number of isolated nodes, and eigenvector centrality (see *SI* for definitions and analysis, Fig. S1).

Initial conditions are not driving the differences we observe: there is no significant difference at the 5% level for the level of cooperation and the mean total payoff between any two treatments in round 1 (see *SI*, Table S5). As a robustness check, we replicate the analysis using a random-coefficient linear regression model based on panel observations controlling for round number and group (SI: Section 5.1). The significant differences in cooperation and payoffs between *S* and the other treatments survive, and, if anything, become stronger as now *S* has significantly higher welfare than *MS* (*p* = 0.01). Similarly, all comparisons above that were insignificant remain insignificant at the 5% level.

Because in all treatments the groups start, on average, from the same level of cooperativeness, it must be that the dynamics of network evolution and the possibility to form strong ties lead to the differences in cooperation that emerge over time. Given that there are no obvious differences in network structure at the aggregate level, we must move to a micro-level analysis to understand the processes that lead to the high cooperativeness and welfare in treatment *S*.

### Individual-level analysis

We begin our investigation by conducting an individual-level analysis of the factors associated with network formation decisions. In particular, we use a random-effects logistic regression model of the likelihood of participants removing, forming, and strengthening ties with standard-errors clustered at the group level, and examine several specifications with different sets of control variables to check for robustness. In addition to the treatment dummy variables, these control variables include network metrics and demographic variables (see *SI* for a more detailed description of these regressors). Differences across treatments are identified using linear combination tests on pairs of estimated regression coefficients for treatment controls and/or interactions of treatment and link strength controls. Hereafter, P values preceded by *LRM* refer to the significance of the regression coefficient while P values preceded by *LCT* refer to the significance of the linear combination test.

The probability to remove a tie is higher when the tie is weak compared to a medium or strong one, and it is particularly high in treatment *S* (see *SI*, Tables S6–S7). In particular, a medium strength tie is less likely to be removed than a weak tie in treatment *M* (LCT, *P* = 0.04). Similarly, a strong tie is less likely to be removed than a weak tie in treatment *S* (LCT, *P* = 0.001). In treatment *S* a weak tie with a defector is more likely to be removed than in *B* (LCT, *P* = 0.001), *M* (LCT, *P* = 0.02), and *MS* (LCT, *P* = 0.04). There are no other statistically significant differences at the 10% level in the probability of removing a weak tie with a defector between any other treatment pair, except for *MS* and *B* (LCT, *P* = 0.02)

While there are no differences across treatments in forming a tie in the first place, participants in treatment *S* are more selective in strengthening a tie. Independently of treatment, two individuals have no information about each other before forming a tie, and therefore it is unsurprising that there is no difference at the 10% level across treatments in the probability of forming a tie (see *SI*, Tables S8–S9) because each participant has, on average, the same willingness to give a chance to another participant. However, once a tie is formed, an individual can see another participant’s last 3 actions when deciding whether to strengthen it. In this case (see *SI*, Tables S10–S11), individuals in *S* are less likely to strengthen a tie than participants in *M* (LRM, *P* = 0.03) and *MS* (LCT, *P* = 0.03).

In summary, participants in *S* adopt what we can dub an “all-or-nothing” strategy to tie formation and removal: they are more selective in strengthening ties and they tend to remove more weak ties as the high rewards available from cooperating with neighbors connected by strong ties make them less tolerant to occasional defectors. The consequences of this all-or-nothing strategy are persistent because strong ties are less likely to be removed than weak ties.

We begin the investigation of the consequences of this strategy for cooperation by using a random-effects logistic regression model of the likelihood of a pair of participants to cooperate (see *SI*, Tables S14–S15). The cooperativeness of a pair of participants is increasing in the strength of their tie, and, in treatment *S* only, a strong tie boosts their cooperativeness compared to its level before they formed the strong tie. When we pool all observations, we observe that both strong and medium ties are more cooperative than weak ties (LCT, *P* = 0.000 and *P* = 0.001) and strong ties are more cooperative than medium ones (LCT, *P* = 0.04). This is unsurprising because participants tend to strengthen ties only with cooperative individuals, but it shows that the all-or-nothing strategy leads to keeping links with cooperators and getting rid of ties with defectors. What is more surprising is that the interaction variable of having a strong tie with cooperativeness level is significant indicating that having a strong tie boosts the cooperativeness of an already rather cooperative partnership (LRM, *P* = 0.001). Running the same regression for each treatment, we see that this cooperativeness boost is driven by treatment *S* (LRM, *P* = 0.001) and it is insignificant for *MS*. We argue that this finding under treatment *S* is not caused by the higher stakes in treatment *S*. The existing studies have shown that increasing the stake size has no impact on cooperative behaviour, in respectively, sequential prisoners’ dilemma and in an n-person public good contribution game^34,35^.

**Figure 3:**
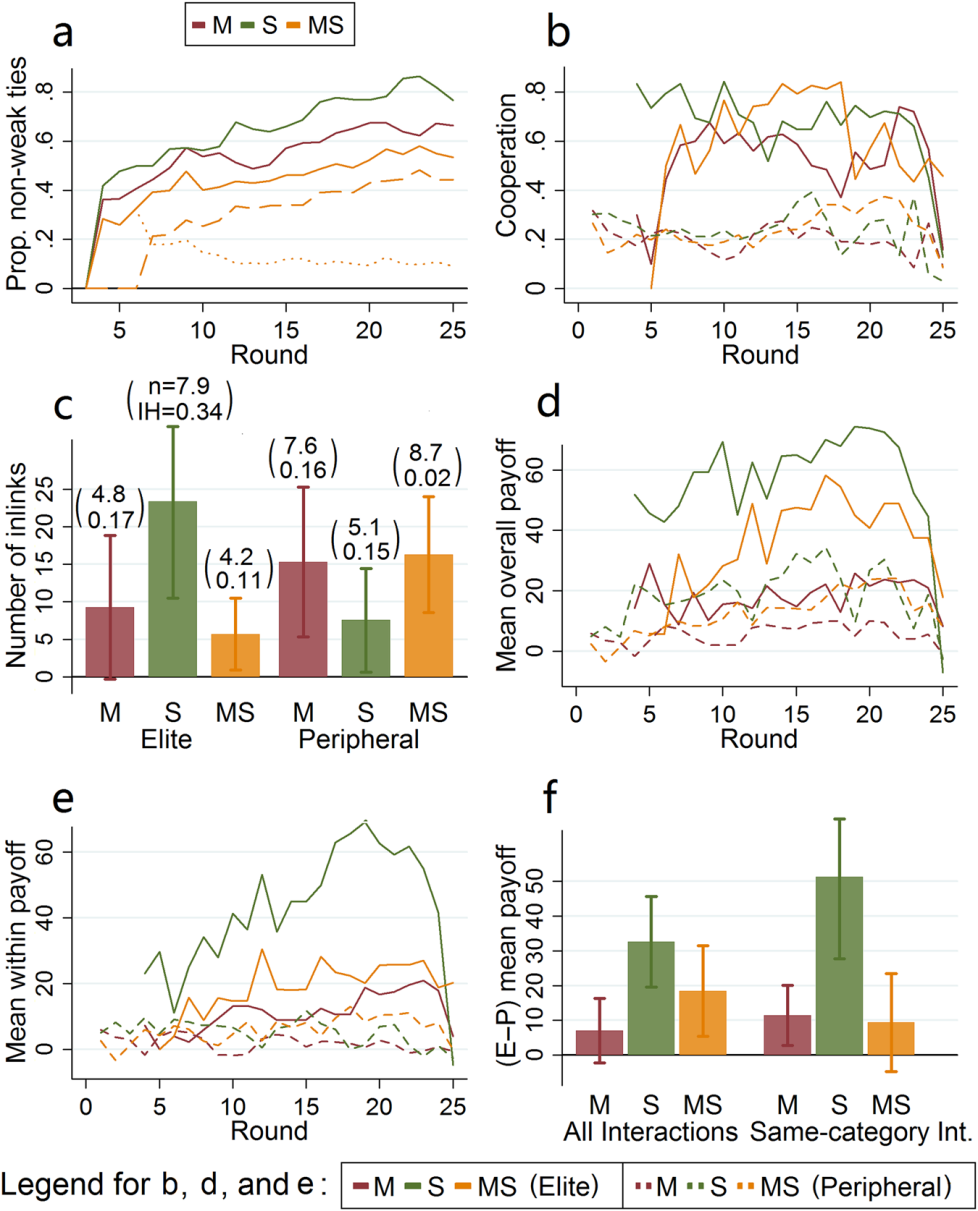
Evolution of the (**a**) proportion of non-weak ties, and (**b**) cooperation level for elite and peripheral participants. (**c**) number of ties among participants of the same category (*inlinks*), where the values on top of the bars refer to the mean size *n* and the inbreeding homophily index (IH). Evolution of (**d**) mean payoff from all interactions and (**e**) payoff from same-category interactions for elite and peripheral participants. (**f**) payoff difference between elite and peripheral participants.

A natural outcome of the all-or-nothing strategy to link formation is that the participants who apply it successfully end up being connected mostly through non-weak ties. Figure 3a shows the evolution of the proportion of non-weak ties in *M*, *S* and *MS*: they start from 0 by design but climb to above 40% in all treatments in the latest rounds. The consequences of the all-or-nothing strategy are mostly present and salient in treatment *S* where the proportion of non-weak ties in the last 8 rounds is above 80%, which is significantly higher than in *M* (*KW*, *P* = 0.03; *DT*, *P* = 0.05) and *MS* (*DT*, *P* = 0.004).

### Elite and peripheral

In order to investigate how the all-or-nothing strategy affects participants’ connections, cooperativeness and payoffs, it is informative to divide participants into two categories. In the first one there are what we dub “elite” participants who have more than 80% of their ties as non-weak. The second category consists of the remaining “peripheral” participants. As the analysis in the SI (Section 4) shows, this categorization is justified by the bimodal nature of the proportion of non-weak ties across participants. Below we explore how this categorization is useful in understanding how a high level of cooperation and welfare emerges in *S* through the formation of a cooperative elite that does not form in the other treatments. We focus on rounds 15–23 to allow for enough time for the emergence of the elite.

A comparison between elite and peripheral individuals within each treatment shows that elite participants are more cooperative (see *SI*, Table S17). Figure 3b plots the evolution over time of the cooperativeness of elite and peripheral individuals for each treatment. Elite individuals are significantly more cooperative than peripheral ones in *S* (Wilcoxon-Mann-Whitney test, WMW hereafter, *P* = 0.01), *M* (WMW, *P* = 0.02), and *MS* (WMW, *P* = 0.04).

There are, however, important differences in the population of elite participants across treatments. Figure 3c shows the number of ties among participants of the same category for elite and peripheral participants across treatments (see *SI*, Tables S17–S19). Elite participants in *S* have on average 23.4 ties with each other which is significantly higher than peripheral ones in the same treatment (7.5; WMW, *P* = 0.05) as well as higher than elite participants in *M* (9.3; KW = 0.009, *P* = 0.007; DT, *P* = 0.007) and *MS* (5.7; DT, *P* = 0.002). In *M* and *MS*, it is actually peripheral participants who have marginally more ties with each other than elite ones (WMW, *P* = 0.09 for *M* and *P* = 0.07 for *MS*). Moreover, peripheral participants in *M* and *MS* have more links with each other than peripheral participants in *S* (KW, *P* = 0.045; DT, *P* = 0.03 and *P* = 0.009).

Two reasons drive the differences between elite and peripheral participants when we compare *S* and the other treatments (see *SI*, Table S18). The first one is that there are on average 7.9 elite participants in *S*, but only 4.8 in *M* (KW, *P* = 0.009; DT, *P* = 0.008) and 4.2 in *MS* (DT, *P* = 0.002). The second one is that elite participants in *S* tend to connect with each other more than elite individuals in *M* and *MS* even after accounting that there are more of them. The *Inbreeding Homophily (IH)* index captures the extent to which individuals of a group tend to connect to each other more than with outsiders with 0 denoting no bias and 1 denoting that they only connect with each other^36^. It is normalized by the size of the group so any differences are not caused by, e.g., the fact that there are more elite individuals in one treatment (see Materials and Methods). As Fig. 3c summarizes, elite individuals in *S* have *IH* = 0.34, which is significantly higher than elite participants in *MS* (*IH* = 0.11; KW, *P* = 0.143; *DT*, *P* = 0.03) and marginally higher than in *M* (*IH* = 0.17; *DT*, *P* = 0.07). Moreover, the only category for which the *IH* index is significantly higher than 0 is elite participants in *S* (*WMW*, *P* = 0.04; see SI, Table S20).

The tendency to connect to each other and the high cooperativeness of elite participants lead to the formation of a cooperative elite in *S* which allows members to earn a significantly higher payoff than elite participants in other treatments (see *SI*, Table S18). Figure 3d shows the evolution of average payoffs for each category divided by treatment. Elite participants in *S* have a higher average payoff than in *M* (KW, *P* = 0.012; DT, *P* = 0.001) and marginally higher than in *MS* (DT, *P* = 0.07). These differences are entirely driven by the highly beneficial interactions within the elite in *S*. Participants belonging to the elite in *S* derive 81% of their overall payoff from interactions with other members of the elite, while this proportion is significantly lower in *M* (47%; KW, *P* = 0.002; DT, *P* = 0.003) and *MS* (41%; DT, *P* = 0.001). Figure 3e shows the evolution of average payoffs from interactions with members of the same category divided by treatment. Elite members in *S* have significantly higher average payoffs from interactions with other elite members than in *M* (KW, *P* = 0.005; DT, *P* = 0.001) and *MS* (DT, *P* = 0.008), and there is no difference between *M* and *MS*.

The outcome is that in treatment *S* there is a large inequality in welfare between members of the elite and participants outside of it which is not present in the other treatments (see *SI*, Table S18). Figure 3f shows the difference in average payoff between elite and peripheral participants for all the interactions (left panel) and the interactions with other participants of the same category only (right panel). In particular, if we consider all interactions then the difference in average payoff between elite and periphery members in *S* is significantly higher than in *M* (KW, *P* = 0.013; DT, *P* = 0.002) and *MS* (DT, *P* = 0.03), and there is no difference between *M* and *MS*. Once we consider the average payoff from interactions with members of the same category only, the difference in average payoff between elite and peripheral members in *S* is significantly higher than in *M* (KW, *P* = 0.003; DT, *P* = 0.004) and *MS* (DT, *P* = 0.001), and there is no difference between *M* and *MS*.

As a robustness check, we replicate the analysis comparing the group of elite participants across treatments using a random-coefficient linear regression model based on panel observations controlling for round number and group (SI: Section 5.1). All the differences in group size, homophily, average payoff and inequality in welfare between the elite in *S* and the other treatments all remain significant at the 5% level.

## Discussion

**Figure 4:**
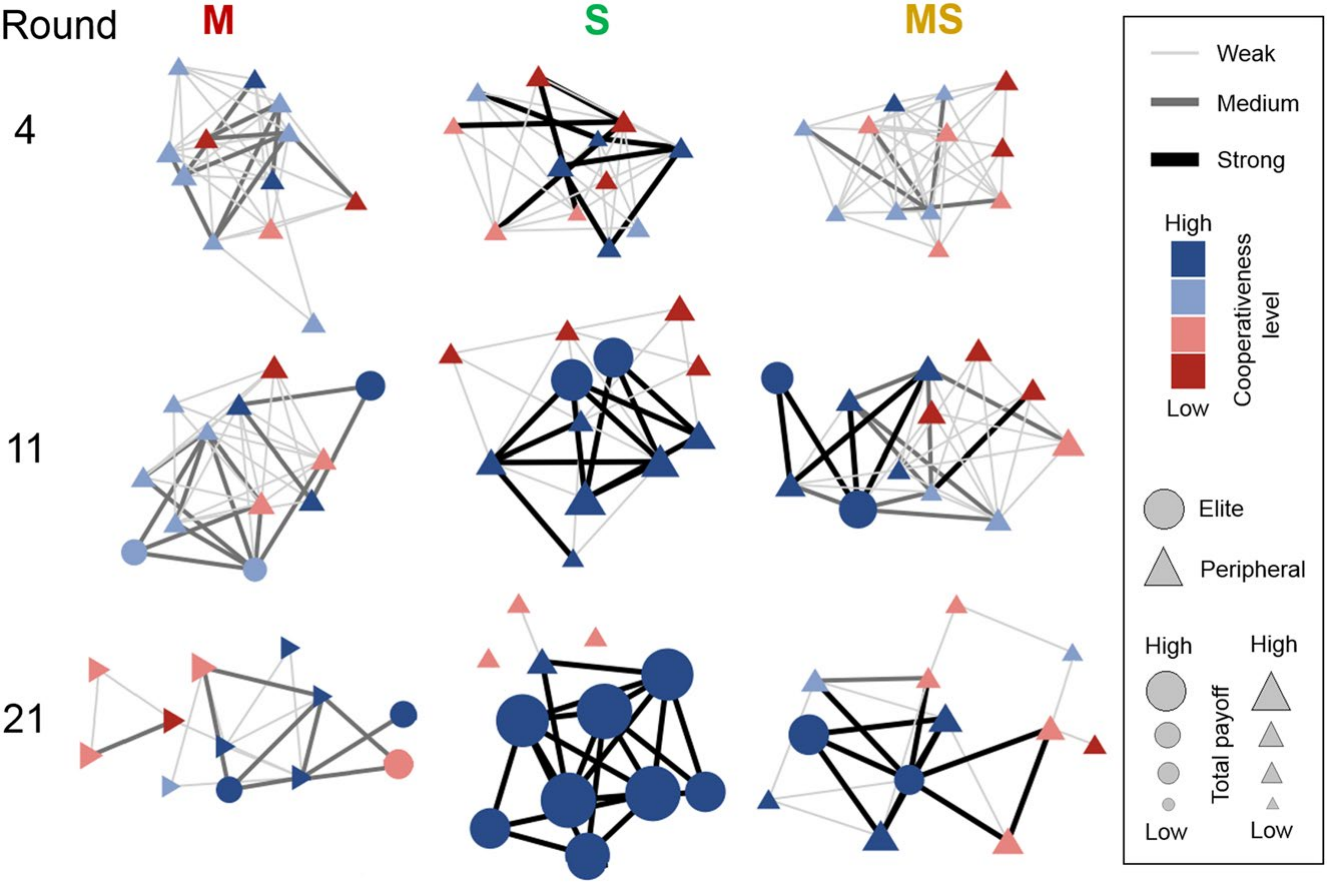
Snapshots from representative experimental sessions to illustrate the evolution of network structure and cooperative behavior. Each column shows one session from each treatment, from left to right: *M*, *S* and *MS*. Each row is a round: 4, 11 and 21 from top to bottom. The node colours correspond to the number of cooperative actions participants have chosen in the previous 3 rounds (cooperativeness level). The shape of the nodels corresponds to the category the node belongs to: circles for elite and triangles for peripheral participants. The size of each node is scaled according to the total payoff it has obtained in the previous 3 rounds. The thickness of ties represents their strength.

Figure 4 provides a visual summary of the results using snapshots from representative sessions for *M*, *S* and *MS*. In the aggregate, the cooperation level and payoffs are higher in *S* compared to *M* and *MS*. This is the result of participants in *S* adopting an all-or-nothing strategy to link formation and removal: the high rewards from mutual cooperation with a neighbor through a strong tie make them more selective in strengthening a tie than participants in *M*/*MS* as well as more likely to remove weak ties because these high rewards make them less tolerant to defectors. This strategy leads to the formation of a numerous, close-knit and highly cooperative elite of strongly connected participants in *S* who amass the bulk of payoffs and marginalize others to a weakly connected periphery. Members of the elite in *S* are better off than the analogous participants in *M* and *MS* where a numerous and close-knit elite fails to form because the different incentives in the network formation process lead to a less frequent utilization of an all-or-nothing strategy to link formation.

The first contribution of this paper is to show the existence of a causal link between the ability to form strong ties and the emergence of cooperation. The result confirms the theoretical predictions from simulation-based studies that strong ties promote cooperative behavior. Furthermore, it validates the prediction that the increase in cooperation is driven by a cluster of cooperator who are closely connected to each other through strong ties^15–18^. The experimental results also highlight how the lack of gradation in strengthening a tie is conducive to the emergence of the cluster of cooperators, which is a variation that has not been explored in the theoretical literature and may be a fertile area for future work.

The second contribution is to discover how strong ties promote cooperation by reinforcing a well-known driver of cooperative behavior. A central mechanism for the emergence of cooperation in dynamic networks is behavioral reciprocity^37–39^ because individuals decide whom to interact with depending on another individual’s past history^25,30^. The all-or-nothing strategy to the strengthening of ties increases participants’ recourse to direct reciprocity because it allows them to enhance the rewards for cooperation and augment the cost of being punished through foregone gains.

The final contribution is to develop a novel methodology to study the role of strong ties in an experimental setting. Up to now there has been no experimental study investigating how the strength of ties causally determine individual behaviour and economic outcomes^33^, leaving untested several theoretical predictions both within^15–18^ and outside the domain of cooperation^40^. Our methodology is directly applicable to investigating the role of strong ties in other games in the same class of games employed in our setup and it has the potential to be extended to studying games outside of this class, which are both avenues for further research.

## Materials and Methods

### Ethics statement

We confirm that all methods in this research were carried out in accordance with the relevant guidelines and regulations. The experimental protocols in this research were approved by the Nanyang Technological University Institutional Review Board (Ref. Code IRB-2015-12-028). The informed consent was obtained from all participants.

We recruited students from Nanyang Technological University (NTU) to participate in the experiments in the NTU Behavioral and Experimental Economics Lab. We ran a pilot with 24 students to ensure that the participants understood the task and the instructions. The sessions for the actual experiment were conducted over 5 consecutive days during the same week: 384 students participated in 16 sessions resulting in 8 groups or networks for each of the 4 treatments. The average earnings per participant was *S*$18.08 (including a *S*$2 fixed fee for participation) and the average duration of an experimental session was two hours. Participants remained completely anonymous throughout the experiment and repeated participation was not allowed. See the *SI* for further details. To ensure that participants understand the tasks they would need to go through, we provide a pre-experiment quiz containing several questions about the experiment tasks. Only when participants have answered all questions correctly that we proceed with the experiment.

The homophily analysis follows the set-up and definitions in^36^, which we briefly summarize here. Suppose there are 2 different categories of individuals *i* = 1, 2. Let *N*_*i*_ denote the number of category *i* individuals in the population and let *w*_*i*_ = *N*_*i*_/*N* be the relative fraction of *i* individuals in the population, where *N* = *N*_1_ + *N*_2_. Let *s*_*i*_ denote the average number of connections that individuals of category *i* have with individuals who are of the same category and let *d*_*i*_ be the average number of connections that category *i* individuals form with individuals of categories different than *i*. The *Homophily index H*_*i*_ is equal to *H*_*i*_ = *s*_*i*_/(*s*_*i*_ + *d*_*i*_) so it captures the fraction of the ties of individuals of category *i* that are with that same category. A drawback of this index is that it varies with population size so it is not possible to use it to evaluate the extent of homophily for categories of individuals belonging to groups of different sizes. *Inbreeding Homophily* is the terminology used in the sociological literature to refer to the tendency of connecting with individuals of the same category beyond the effect of relative group size^41^. Let us define a profile (*s*_1_, *d*_1_, *s*_2_, *d*_2_) to satisfy baseline homophily if *H*_*i*_ = *w*_*i*_ and inbreeding homophily if *H*_*i*_ > *w*_*i*_. The *Inbreeding Homophily index* of category *i* is defined as *IH*_*i*_ = (*H*_*i*_ − *w*_*i*_)/(1 − *w*_*i*_), and it measures the amount of bias with respect to baseline homophily as it relates to the maximum possible bias which is the denominator 1 − *w*_*i*_. It can be easily checked that we have inbreeding homophily for category *i* if and only if *IH*_*i*_ > 0. The index of inbreeding homophily is zero if there is pure baseline homophily and is one if a group completely inbreeds.

## Supplementary information

Supplementary Info
