# Supplementary material for: Strong links promote the emergence of cooperative elites
Source: Sci Rep. 2019 Jul 26;9:10857. doi: 10.1038/s41598-019-47278-2 (PMC6659657; doi:10.1038/s41598-019-47278-2)
Supplement: Supplementary file 1 — Supplementary Info [file 41598_2019_47278_MOESM1_ESM.pdf]

Strong links promote the emergence of cooperative elites: Supplementary Information

Edoardo Gallo[1], Yohanes E. Riyanto[2], Tat-How Teh[3], Nilanjan Roy[4]

[1]Faculty of Economics, University of Cambridge, Sidgwick Avenue, Cambridge CB3 9DD, UK and Queens' College, CB3 9ET, UK.
[2]Division of Economics, School of Social Sciences, Nanyang Technological University, 14 Nanyang Drive HSS 04-70, Singapore.
[3]Department of Economics, Faculty of Arts and Social Sciences, National University of Singapore, AS2 06-02 1 Arts Link, Singapore.
[4]Department of Economics and Finance, College of Business, City University of Hong Kong, 83 Tat Chee Avenue, Kowloon Tong, Hong Kong.

June 21, 2019

1 Experimental details

The experiment was conducted at the NTU Behavioral and Experimental Economics lab and is programmed using Z-Tree [1]. It has three parts. Part 1 consists of 2 stages. In Stage 1 participants play a dictator game. Each of them is assigned the role of an allocator and is asked to divide an endowment of 100 experimental currency units (ECUs) between himself (herself) and an unknown recipient from the *next* experimental session. In stage 2, each of them acts as a recipient of a division proposed by a randomly selected allocator from a previously conducted experimental session. Participants can never be beneficiaries of their own allocations nor allocations made by their recipients in Stage 1 in order to be able to isolate the effect of reciprocity on the allocation decision.

Part 2 is the main part of the experiment. Participants play the same game for 25 rounds, and thereafter there is a 50% chance that they proceed to a new round. There are three stages in each round.

In stage 1, participants can propose and (or) remove ties with other participants, and they have 70 seconds to make their decisions. In the initial round there are no ties among any of the participants. A participant can propose a tie to any of the other participants, and the decision whether the tie is formed depends on the recipient's acceptance/rejection of the proposal in stage 2 (see below). After the first round, in stage 1 a participant can also unilaterally remove any of their ties from the previous round. For each participant, the interface displays a visualization of their ties to their neighbors (see section 6), and the nodes are coloured in either green or blue depending on whether their last action in the game was to cooperate or defect respectively.

In stage 2, participants are prompted to accept or reject the ties other participants proposed in stage 1. Notice that if two participants send a tie proposal to one another in stage 1, then the tie is automatically formed and there will be no prompt given at this stage. Again, tie removals are unilateral so the tie removal decisions in stage 1 are implemented automatically.

In stage 3, participants play a Prisoner's Dilemma (PD) game with all their neighbours. They must choose either action A or action B. The former and the latter correspond to, respectively, cooperation (C) and defection (D). The chosen action will be played against all neighbors with whom the participant

is connected to. If a participant is not connected to other participants, and thus has no neighbors, this participant will have no action to choose. Once all participants make their choices, they see the points they receive from the game with each of the other participants. They receive zero points in this stage from participants with whom they are not connected to. They will also receive a reminder of their chosen action and the action chosen by their neighbors.

With the exception of the baseline treatment, participants also have the possibility to strengthen their ties. In stage 1 a participant who has been linked to another participant for at least three consecutive rounds can propose to strengthen the tie. In stage 2, participants are prompted to accept or reject the tie strengthening proposals made by other participants in stage 1. The removal of strengthened ties in stage 1 follows the same procedure as the removal of weak ties. That is, each participant can unilaterally remove a tie independently of the strength of the tie.

Participants' earnings from this part are based on 6 randomly selected rounds. For each participant, two other participants are randomly picked for each of the selected rounds and the sum of the points won in the interaction with these 12 randomly picked participants constitutes the total sum of points won by the participant in this part. Notice that it is possible to pick an unconnected pair of participants and, in that case, the earnings from that interaction are equal to zero.

Part 3 elicits participants' risk preferences using a version of the multiple price lottery (MPL) method proposed by [2]. In particular, participants are shown 10 pairs of choices, where in each pair they have to decide their choice between the two available options A and B. Option A always gives a fixed amount as a payoff while option B has varying expected payoffs. Specifically, option A always gives 20 ECU, while option B gives either 60 ECU or 0 ECU with varying probabilities. In the earlier pairs, option B has a low probability of yielding 60 ECU, but as we move down the pairs this probability increases. One pair is randomly picked for payment so if they choose option A in the randomly selected pair they receive 20 ECU, and if they instead choose option B they receive either 60 ECU or 0 ECU with the probabilities stated in that randomly selected pair.

After the experiment, participants are asked to fill in a demographic survey and to answer a standard interpersonal trust question taken from the World Values Survey (WVS): *"Generally speaking, would you say that most people can be trusted or that you need to be very careful in dealing with people?"* The choice is between *"Most people can be trusted"* and *"You cannot be too careful"*.

We conduct 4 treatments to evaluate different dimensions of the strength of ties and their impact on the emergence of cooperation. In the baseline (B) treatment, participants can only choose whether or not to be connected by a *weak* tie with other participants. The points they obtain from the PD game are summarized in Table S1.

Table S1: **Payoff Table - Weak Tie Strength (all treatments)**

	Neighbor		
You		C	D
	C	3	-5
	D	5	-3

If a participant plays C and her neighbor plays C, each will receive 3 points. However, if her neighbor plays D instead, she will receive -5 points and her neighbor will receive 5 points. If she plays D and her neighbor plays D, each will receive -3 points. A participant's choice of either C or D is implemented against all neighbors.

In the Medium (M) treatment participants can connect with other participants by a weak tie and play the game in Table S1, and they can also strengthen their weak ties with other participants by forming medium strength ties with twice the strength of weak ties. We represent the strength of a tie by applying a scaling factor s to the payoffs of the game and assuming a one-to-one relation between the strength of

the tie and the scaling factor. In treatment M this means that $s = 2$ so Table S2 shows the payoffs for the PD game between two participants linked by a medium tie strength.

Table S2: **Payoff Table - Medium Tie Strength (treatments M and MS)**

Neighbor			
You		C	D
	C	6	-10
	D	10	-6

The strong (S) treatment is the same as M, but the scaling factor is $s = 4$. Table S3 shows the payoffs for the PD game between two participants linked by a strong tie.

Table S3: **Payoff Table - Strong Tie Strength (treatments S and MS)**

Neighbor			
You		C	D
	C	12	-20
	D	20	-12

Finally, in the medium and strong ties MS treatment, participants can be connected to their neighbors by weak ($s = 1$), medium ($s = 2$), and strong ($s = 4$) ties. Notice that the option to strengthen from $s = 1$ directly to $s = 4$ is only available in the S treatment.

All the participants are undergraduate students at Nanyang Technological University (NTU) studying different majors ranging from the sciences to the humanities. We first ran a pilot experiment with 24 students to ensure that participants understood the task and instructions. The sessions for the actual experiment were conducted over 5 consecutive days during the same week. In total, 384 students are randomly allocated to 16 experimental sessions so there are 4 sessions for each of the 4 treatments. Each session consists of 2 groups of size 12 for a total of 8 independent observations at the network level per treatment. The choice of a group size of 12 ensures that the network is large enough to allow the emergence of interesting structural features while at the same time it is small enough to have a good number of independent observations per treatment. The chosen group size is also consistent with previous studies on cooperation in dynamic networks [3, 4, 5, 6, 7].

The average earnings per participant is SGD 18.08 (including a SGD 2 fixed fee for participation), which is equivalent to around USD 13 at the time of the experiment.[1] The average duration of an experimental session is 2 hours. Participants are only allowed to participate in 1 session of the experiment and they remain completely anonymous throughout the experiment. Out of the 384 participants, 55.2% are female, 67.2% are Singaporeans, and the average age is 21.2 years. About 23% of the participants have had some prior exposure to Game Theory, and approximately half of the participants had participated in at least one laboratory experiment previously. From the World Values Survey (WVS) interpersonal trust question, 21% believe that others can be trusted. In the post-experiment questionnaire almost all participants state that they had no problem in understanding the experiment.

2 Statistical analysis

In this section we describe the technical details and provide further statistical analysis of our data. We organize this section into three sub-sections: Section 2.1 presents the analysis at the aggregate (session)

[1] The hourly earning from a part-time job in Singapore is approximately SGD 7, so the average earnings in the experiment are significantly higher than the average hourly earning from a part-time job.

level, Section 2.2 presents the regression analysis at the individual level, and Section 2.3 presents the analysis at the category level (elite and peripheral groups).

2.1 Aggregate level

We use the Kruskal-Wallis test [8] to detect treatment effects, and use the Dunn's test [9] as the post-hoc test for multiple pairwise comparisons between treatments. We choose the Dunn's test because: (a) it uses the same ranks as those in the Kruskal-Wallis test; and (b) it uses the pooled variance implied by the null hypothesis in the Kruskal-Wallis test. Other pairwise comparison tests, e.g. the Mann-Whitney test, are less appropriate to be used as the post-hoc test following the Kruskal-Wallis test because they violate points (a) and (b) above. Notice that we have a small sample ($n = 8$) per treatment after aggregation at the network level so even a 10% significance level is suggestive of a sizable treatment effect given the small number of data points.

Table S4 lists the p-values obtained from the Kruskal-Wallis tests and the Dunn's tests done on various aggregate-level variables. The aggregate level variables are the following:

- *Cooperation*: The number of pairs with mutual cooperation at the end of the round, divided by the total number of possible pairs in a complete network. There are 66 possible pairs for a network with 12 nodes.
- *Cooperation (Non-weak)*: The number of non-weak pairs with mutual cooperation at the end of the round, divided by the total number of possible pairs in a complete network (66 pairs).
- *Number of Ties*: The total number of ties that are present at the end of the round.
- *Payoff*: The average number of points obtained by participants from all interactions at the end of the round.
- *Average Local Clustering*: For each node, the local clustering coefficient measures how connected the node's neighbors are, and it is formally defined as :

$$C_i = \frac{2\sum_{j,k\in N_i} g_{ij}g_{ik}g_{jk}}{d_i(d_i-1)} \tag{1}$$

  where $g_{ij} = 1$ if a tie exist between i and j and zero otherwise, $N_i = \{j|g_{ij} = 1\}$, and $d_i = |N_i|$. We then take the average coefficient value over all 12 nodes in the network.
- *Proportion of Non-weak Ties*: The proportion of the *Number of Ties* in the network that are non-weak ties.
- *Number of Isolated Nodes*: The number of nodes with zero ties.
- *Number of Components*: The number of disjoint path-connected subgraphs when the network is not connected. Each isolated node counts as a component.
- *Eigenvector Centrality*: For each node, eigenvector centrality measures the influence of the node in the network. For a given network g, the centrality of a node, C_i^e, is proportional to the sum of the centrality of its neighbors, i.e $\lambda C_i^e = \Sigma_j g_{ij} C_j^e$. In matrix notation,

$$\lambda C^e = gC^e. \tag{2}$$

  Thus, C^e is an eigenvector of g, and λ is its corresponding eigenvalue. The centrality measure is then averaged over all 12 nodes in the network.

We report the results for the same range of rounds as those in the main text, which is rounds 8 to 23. The only exception is the proportion of strong ties which, as in the main text, is reported from rounds 15 to 23. This is because strong ties take time to form and so to capture this we focus on the later rounds.

Table S5 replicates the analysis in Table S4 for round 1 only. The results show that in the very first round there are no statistically significant differences at the 5% level in our aggregate-level variables across treatments. This is reassuring because it shows that eventual differences across treatments are not driven by path-dependence due to a bias in the network formed in the first round. Note that we do not include the last five aggregate level variables from Table S4 because they are either not well-defined or not meaningful in the first round.

Table S4: The results of the Kruskal-Wallis tests and the Dunn's tests for different variables at the aggregate level from round 8 to 23. We have $n = 8$ sample size per treatment after aggregation at the group (network) level.

**Variables**	**Kruskal-Wallis Tests** $\binom{\chi^2(3)}{P\text{-value}}$	**Dunn's Test** $\binom{z\text{-stat}(col-row)}{P\text{-value}}$			
Cooperation	3.779<br>0.286		B	M	S
		M	0.053<br>0.479		
		S	-1.652<br>0.049	-1.706<br>0.044	
		MS	-0.426<br>0.335	-0.479<br>0.316	1.226<br>0.110
Cooperation (Non-weak)	4.865<br>0.088		M	S	
		S	-1.556<br>0.060		
		MS	0.283<br>0.389	1.838<br>0.033	
Number of Ties	2.862<br>0.413		B	M	S
		M	0.759<br>0.224		
		S	-0.279<br>0.389	-1.039<br>0.149	
		MS	0.122<br>0.110	0.466<br>0.321	1.506<br>0.066
Payoff	12.145<br>0.007		B	M	S
		M	-0.719<br>0.236		
		S	-3.278<br>0.001	-2.558<br>0.005	
		MS	-1.759<br>0.039	-1.039<br>0.149	1.519<br>0.064

Table S4 (Continued)

**Variables**	**Kruskal-Wallis Tests** $\binom{\chi^2(3)}{P\text{-value}}$	**Dunn's Test** $\binom{z\text{-stat}(col-row)}{P\text{-value}}$			
Average Local Clustering	3.855<br>0.277		B	M	S
		M	1.092<br>0.137		
		S	-0.692<br>0.244	-1.785<br>0.037	
		MS	0.773<br>0.219	-0.319<br>0.375	1.465<br>0.071
Proportion of Non-weak Ties (T15-T23)	6.980<br>0.031		M	S	
		S	-1.629<br>0.050		
		MS	0.989<br>0.161	2.616<br>0.004	
Number of Isolated Nodes	1.573<br>0.665		B	M	S
		M	-0.986<br>0.161		
		S	-0.027<br>0.489	0.959<br>0.169	
		MS	-0.824<br>0.205	0.162<br>0.436	-0.796<br>0.213
Number of Components	1.076<br>0.778		B	M	S
		M	-0.726<br>0.234		
		S	-0.013<br>0.495	0.713<br>0.238	
		MS	-0.767<br>0.222	-0.040<br>0.484	-0.753<br>0.226
Eigenvector Centrality	4.713<br>0.194		B	M	S
		M	0.453<br>0.325		
		S	-0.267<br>0.395	-0.720<br>0.236	
		MS	1.732<br>0.042	1.279<br>0.101	1.999<br>0.023

Table S5: The results of the Kruskal-Wallis tests and the Dunn's tests for different variables at the aggregate level for round 1. We have $n = 8$ sample size per treatment after aggregation at the group (network) level.

**Variables**	**Kruskal-Wallis Tests** $\binom{\chi^2(3)}{p\text{-value}}$	**Dunn's Test** $\binom{z\text{-stat}(col-row)}{p\text{-value}}$	B	M	S
Cooperation	1.295<br>0.730	M	-0.093<br>0.463		
		S	-0.573<br>0.283	-0.480<br>0.315	
		MS	0.560<br>0.288	-0.653<br>0.257	1.133<br>0.128
Number of Ties	3.150<br>0.369	M	1.605<br>0.054		
		S	1.106<br>0.134	-0.499<br>0.308	
		MS	0.149<br>0.067	-0.108<br>0.457	0.391<br>0.348
Payoff	0.702<br>0.872	M	0.373<br>0.354		
		S	0.507<br>0.306	0.133<br>0.447	
		MS	0.827<br>0.204	0.453<br>0.325	0.320<br>0.374
Average Local Clustering	2.613<br>0.455	M	1.510<br>0.065		
		S	1.149<br>0.125	-0.361<br>0.359	
		MS	1.189<br>0.117	-0.321<br>0.374	0.040<br>0.484

The above two tables include the results from a comparison across treatments of several aggregate level variables that we do not discuss in the main text. Figure S1 is the equivalent of Figure 2 in the main text for some of these additional variables. In particular, it shows the evolution of the average local clustering coefficient (Figure S1A), the average eigenvector centrality (Figure S1B), the number of isolated nodes (Figure S1C), and the number of components in the network (Figure S1D). Table S4 shows that in most cases there are no differences in these network metrics at the 10% significance level across all treatments. The only exceptions are: average local clustering is higher in treatment S than M (DT, $p < 0.05$) and eigenvector centrality is lower in treatment MS than B and S (DT, $p < 0.05$).

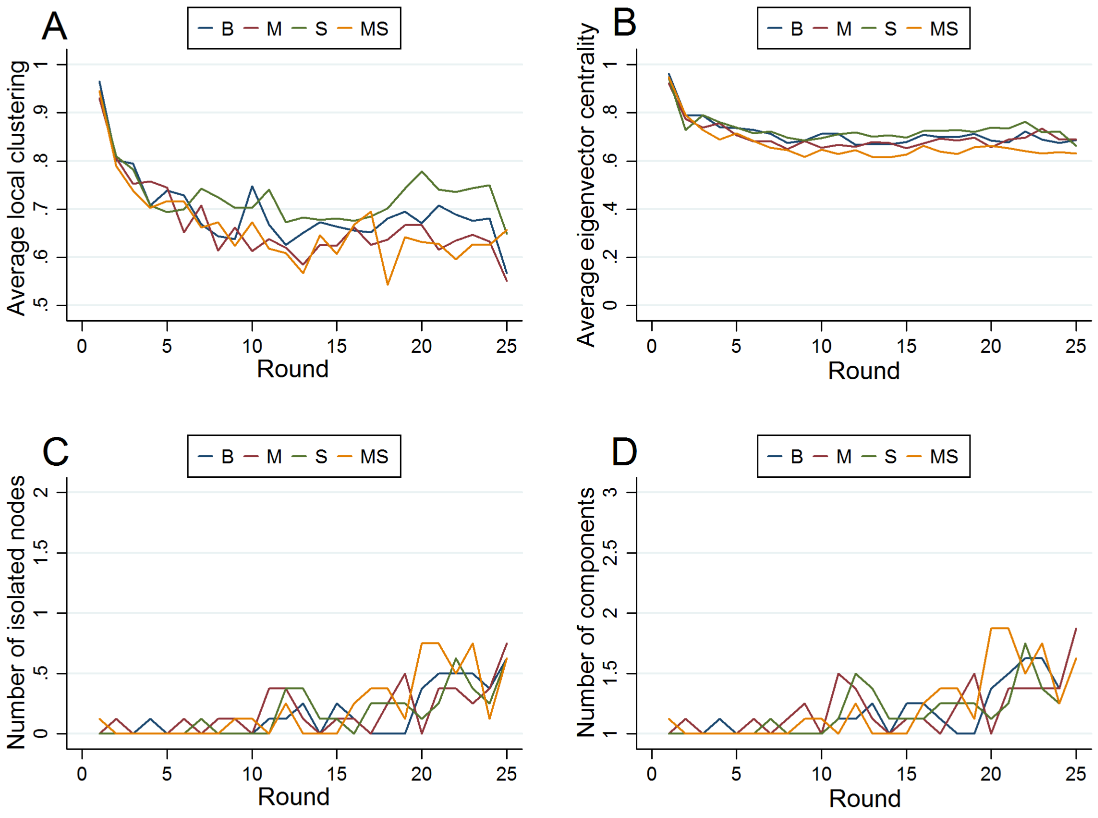

Figure S1: The evolution of selected network metrics over 25 rounds for each treatment. Panel A depicts the average local clustering coefficient across treatments. Panel B shows the average eigenvector centrality across treatments. Panel C shows the number of isolated nodes across treatments. Panel D presents the number of components across treatments.

2.2 Individual level

Table S6 presents the results of random-effects logit regressions using observations from rounds 8 to 23. The dependent variable is the tie removal decision made by participants, which is equal to 1 if the tie is unilaterally removed. In all of these regressions we cluster the standard errors at the group level. We exclude observations where the linked partner cooperated in the last round prior to the tie removal decision because virtually no participant removes such ties (less than 0.1% of the total observations).

In the regression specification (1), we only include interaction variables between the dummy variables for treatments (M, S, MS) and tie strength (Weak, Medium, Strong). The baseline for comparison is the weak tie in Treatment B. Specification (2) includes a measure of other-regarding behavior of the participant elicited from the dictator game administered at the beginning of the experiment (*Offer Proposer*), and *Other Control Variables* consisting of *Age*, *Gender*, the measure of *Trust* obtained by asking the question taken from the World Values Survey (see section 1) to participants, the switching point in the Holt-Laury risk preference elicitation (*Risk Aversion*), and an indicator variable capturing whether participants have taken a game theory course before (*Game Theory*). For the sake of brevity, in Table S6 we only indicate whether the set of control variables is present in our regressions.

In specification (3), we include a variable capturing each participant's action in the previous round (*Own Last Action*), which equals to 1 if the participant cooperated and 0 if the participant defected, and the number of times the participant cooperated in the last three rounds (*Own Last 3 Actions*). In specification (4), we replace *Own Last Action* and *Own Last 3 Actions* with three network metrics: *Degree*, which is the participant's number of ties, *Local Clustering*, which is the participant's local clustering coefficient, and *Frac. of Non-weak Ties*, which is the participant's proportion of non-weak ties out of all her ties. Finally, in specification (5) we include all the independent variables.

Using the regression results under specification (5) shown in Table S6, we conduct the linear combinations tests (LCT hereafter) on the estimated coefficients of the treatment/tie strength dummy variables. Essentially, LCT verify whether the pairwise differences between these estimated regression coefficients are statistically significant. In Table S7, we report the corresponding p-values based on the null hypothesis stating that the said differences are zero.

Table S6: Random-effects logit regressions of the likelihood of participants removing ties, with the standard errors (SE) clustered at the group (network) level. Observations where the tied partner cooperated in the last round prior to the tie removal decision are excluded.

	(1)	(2)	(3)	(4)	(5)
**Dependent Variable: Tie Removal Decision**					
(M) x (Weak Tie)	0.256	0.259	0.327	0.005	0.279
	(0.449)	(0.435)	(0.403)	(0.379)	(0.365)
(M) x (Medium Tie)	-0.272	-0.313	-0.345	-0.655	-0.278
	(0.488)	(0.480)	(0.470)	(0.443)	(0.458)
(S) x (Weak Tie)	1.159***	1.171***	1.048***	0.946**	1.053***
	(0.331)	(0.342)	(0.302)	(0.291)	(0.264)
(S) x (Strong Tie)	0.097	0.114	-0.023	-0.089	0.220
	(0.373)	(0.354)	(0.325)	(0.346)	(0.326)
(MS) x (Weak Tie)	0.772*	0.730*	0.666*	0.488	0.583*
	(0.338)	(0.335)	(0.287)	(0.285)	(0.259)
(MS) x (Medium Tie)	0.416	0.406	0.252	0.044	0.271
	(0.421)	(0.412)	(0.392)	(0.408)	(0.402)
(MS) x (Strong Tie)	0.012	-0.023	-0.114	-0.377	-0.043
	(0.578)	(0.565)	(0.422)	(0.566)	(0.460)
Own Last Action			0.123		0.070
			(0.110)		(0.117)
Own Last 3 Actions			1.393***		1.490***
			(0.246)		(0.265)
Degree				-0.031	-0.057
				(0.034)	(0.033)
Local Clustering				-1.343***	-1.109***
				(0.279)	(0.297)
Frac. of Non-weak Ties				0.346	-0.300
				(0.345)	(0.333)
Offer Proposer		0.014***	0.015***	0.015***	0.015***
		(0.004)	(0.003)	(0.004)	(0.004)
Round	0.024	0.023	0.020	0.017	0.016
	(0.012)	(0.012)	(0.013)	(0.011)	(0.012)
Other Control Variables	No	Yes	Yes	Yes	Yes
Constant	Yes	Yes	Yes	Yes	Yes
N	9473	9473	9369	9473	9369

Standard errors are in parentheses

* $p < 0.05$, ** $p < 0.01$, *** $p < 0.001$

Other Control Variables include *Age, Female, Trust, Risk Aversion*, and *Game Theory*.

Table S7: Linear combination tests on pairs of estimated regression coefficients based on estimation results under specification (5) shown in Table S6. The tests are conducted to detect whether there are statistically significant within-treatment and between-treatment differences in participants' decisions to remove ties.

Comparison	Difference	P-value
Differences between tie strengths, conditional on treatments		
$[\text{ Medium}-\text{Weak }]_M$	-0.556	0.037
$[\text{ Strong}-\text{Weak }]_S$	-0.833	0.001
$[\text{ Medium}-\text{Weak }]_{MS}$	-0.312	0.257
$[\text{ Strong}-\text{Weak }]_{MS}$	-0.626	0.098
$[\text{ Strong}-\text{Medium }]_{MS}$	-0.314	0.395
Differences between treatments, conditional on having a weak tie		
$M - B$	0.278	0.445
$S - B$	1.053	0.001
$S - M$	0.774	0.024
$S - MS$	0.469	0.035
$MS - B$	0.583	0.024
$MS - M$	0.262	0.349

Here we only focus on specification (5) in Table S6 but the qualitative insights are the same for the regression results using other specifications.

Table S8 shows the results of random-effects logit regressions of the likelihood that a tie is formed. All observations are from rounds 8 to 23. Note that the unit of observations in this regression (and also in regression results shown in Tables S10, S12, and S14 below) is at the pairwise level because of the bilateral nature of the tie formation decision. That is, both must agree to form a new tie. The dependent variable is an indicator variable that equals to 1 if the pair of participants agree to form a tie, and 0 if the pair disagree.

In specification (1) we include the treatment dummies (M, S and MS), and two additional dummy variables capturing the tied partners' last round of action pair: *CD/DC*, which equals to 1 if one of the tied partners cooperated in the last round while the other did not, and 0 otherwise, and *DD*, which equals to 1 if both tied partners defected in the last round and 0 otherwise. Thus, from the specifications of dummy variables, the comparison baseline is the case where both participants in the pair mutually cooperated (*CC*) in the last round in treatment B. Specification (2) and (3) add a host of network metrics (*Local Clustering*, *Degree*, *Fraction of Non-weak Ties*) and additional control variables. Since every observation consists of two participants forming a tie pair, for each of the non-binary variables we include both the *Mean* value and the absolute *Difference* value of the variable for each pair of participants. Similarly, we include two dummies for the binary variables: *Both*, which equals to 1 if the variable takes the value of 1 for both participants, and *One*, which equals to 1 if the variable takes the value of 1 for exactly one participant in the pair. For brevity, we only indicate whether these control variables are present in the regression analysis.

Table S8: Random-effects logit regressions of the determinants of the decision of a pair of participants to form a tie with standard errors (SEs) clustered at the group (network) level.

	(1)	(2)	(3)
**Dep. Var: Tie Formed**			

Treatment M	-0.475	-0.502	0.270
	(0.286)	(0.266)	(0.263)
Treatment S	-0.551	-0.520	0.294
	(0.296)	(0.288)	(0.277)
Treatment MS	-0.352	-0.323	0.346
	(0.257)	(0.241)	(0.243)
CD/DC	0.039	0.028	-0.018
	(0.077)	(0.079)	(0.082)
DD	0.749***	0.728***	0.598***
	(0.147)	(0.145)	(0.132)
Local Clustering (Mean)			-0.216
			(0.198)
Local Clustering (Diff.)			-0.196
			(0.118)
Degree (Mean)			0.083
			(0.045)
Degree (Diff.)			0.006
			(0.025)
Frac. of Non-weak Ties (Mean)			-1.096***
			(0.245)
Frac. of Non-weak Ties (Diff.)			-0.525**
			(0.169)
Offer Proposer (Mean)		0.000	0.001
		(0.004)	(0.004)
Offer Proposer (Diff.)		-0.001	-0.001
		(0.002)	(0.002)
Round	-0.067***	-0.067***	-0.060***
	(0.011)	(0.011)	(0.010)
Other Control Variables	No	Yes	Yes
Constant	Yes	Yes	Yes
N	16506	16506	16485

Standard errors in parentheses

* $p < 0.05$, ** $p < 0.01$, *** $p < 0.001$

Other Control Variables include *Age, Female, Trust, Risk Aversion*, and *Game Theory*.

Table S9: Linear combination tests to evaluate between-treatment differences in the likelihood of a pair of participants to form a tie based on the estimated regression coefficients obtained under each regression specification shown in Table S8.

	Specification (1)		Specification (2)		Specification (3)	
Between-treatment Comparison	Difference	P-value	Difference	P-value	Difference	P-value
$S - M$	-0.076	0.763	-0.018	0.942	0.024	0.901
$MS - M$	0.123	0.540	0.179	0.349	0.076	0.637
$S - MS$	-0.199	0.345	-0.197	0.362	-0.052	0.768

Table S10 presents the results of random-effects logit regressions of the likelihood of a tie strengthening being realized. All observations are from rounds 8 to 23 of treatments M, S, and MS. Round 8 is the earliest round in which a strong tie can form in treatment MS and therefore it provides a natural starting point for the regression analysis. The dependent variable equals to 1 if both tied partners agree to strengthen their tie, and 0 otherwise. We focus on the observations where there is at least two times of mutual cooperations achieved by tied partners in the last three rounds ("Number of CC $\geq$ 2"). This is because the probability of upgrading ties is very low for the observation with Number of CC strictly lower than two in all treatments.

In all specifications we use the weak ties in treatment M as our baseline for comparison. We include the treatment dummies (S and MS), and the number of rounds (among the last three rounds) where both tied partners mutually cooperated (*Number of CC*). All other independent variables included in the regressions are the same as those included in Table S8.

Table S10: Random-effects logit regression of the determinants of pairwise decision to strengthen ties with standard errors (SEs) clustered at the group (network) level, conditional on the observations with at least two past mutual cooperations between partners

	(1)	(2)	(3)
**Dep. Var: Strengthening Realized**			
Treatment S	-1.272	-1.307*	-1.078*
	(0.679)	(0.609)	(0.497)
Treatment MS	0.188	0.145	0.424
	(0.741)	(0.668)	(0.508)
Number of CC	3.929**	3.994**	4.235***
	(1.214)	(1.400)	(0.672)
Local Clustering (Mean)			0.920
			(1.387)
Local Clustering (Diff.)			0.718
			(1.726)
Degree (Mean)			-0.222
			(0.140)
Degree (Diff.)			-0.009
			(0.118)
Frac. of Non-weak Ties (Mean)			-2.732***
			(0.772)
Frac. of Non-weak Ties (Diff.)			-1.918
			(1.012)

Offer Proposer (Mean)		0.015	0.011
		(0.020)	(0.017)
Offer Proposer (Diff.)		0.000	-0.006
	(0.006)	(0.012)	(0.011)
Round	-0.008	-0.015	-0.029
	(0.066)	(0.066)	(0.061)
Other Control Variables	No	Yes	Yes
Constant	Yes	Yes	Yes
N	642	642	642

Standard errors are in parentheses

* $p < 0.05$, ** $p < 0.01$, *** $p < 0.001$

Other Control Variables include *Age, Female, Trust, Risk Aversion*, and *Game Theory*.

Table S11: Linear combination tests to evaluate between-treatment differences in the likelihood of tied partners to strengthen their ties based on the estimated regression coefficients obtained under each regression specification shown in Table S10.

	Specification (1)		Specification (2)		Specification (3)	
Between-treatment Comparison	Difference	P-value	Difference	P-value	Difference	P-value
$S - M$	-1.271	0.061	-1.307	0.007	-1.078	0.030
$MS - M$	0.188	0.800	0.145	0.828	0.424	0.404
$S - MS$	-1.460	0.034	-1.452	0.026	-1.502	0.026

As an additional robustness check, in Table S12 we run a similar regression analysis to the one presented in Table S10, but with two additional dummy variables capturing the tied partners' last round of action pair: *CD/DC*, which equals to 1 if one of the tied partners cooperated in the last round while the other did not, and 0 otherwise, and *DD*, which equals to 1 if both tied partners defected in the last round and 0 otherwise. We further add interaction variables between these dummy variables and the treatment dummy variables. Thus, the baseline of comparison is the case where both tied partners mutually cooperated (*CC*) in the last round in treatment M. Based on the coefficient estimates from the regression analysis shown in Table S12, we run linear combination tests to evaluate whether the differences in the likelihood of tied partners to strengthen their ties are statistically significant between pairs of treatments. The results are reported in Table S13.

Table S12: Random-effects logit regressions of the determinants of pairwise decisions to strengthen ties with standard errors (SEs) clustered at the group (network) level, conditional on the last round action pairs.

	(1)	(2)	(3)
**Dep. Var: Strengthening Realized**			
Treatment S	0.072	-0.177	-0.118
	(0.964)	(0.877)	(0.879)
Treatment MS	0.545	0.499	0.449
	(1.065)	(0.919)	(0.912)
CD/DC	-2.867***	-2.839***	-2.733***
	(0.878)	(0.801)	(0.788)
DD	-3.068***	-3.050***	-2.953***
	(0.876)	(0.799)	(0.781)
(S) x (CD/DC)	-0.984	-0.857	-0.958
	(1.014)	(0.890)	(0.870)
(S) x (DD)	0.065	0.134	0.094
	(1.474)	(1.384)	(1.368)
(MS) x (CD/DC)	-0.542	-0.500	-0.499
	(1.133)	(0.999)	(0.974)
(MS) x (DD)	-1.176	-1.152	-1.104
	(1.455)	(1.265)	(1.223)
Local Clustering (Mean)			-0.775
			(0.587)
Local Clustering (Diff.)			1.772**
			(0.688)
Degree (Mean)			0.060
			(0.089)
Degree (Diff.)			-0.112
			(0.060)
Frac. of Non-weak Ties (Mean)			1.168*
			(0.539)
Frac. of Non-weak Ties (Diff.)			-0.956**
			(0.367)
Offer Proposer (Mean)		0.002	0.001
		(0.012)	(0.012)
Offer Proposer (Diff.)		-0.001	0.000
		(0.006)	(0.006)
Round	0.026	0.023	0.015
	(0.022)	(0.023)	(0.026)
Other Control Variables	No	Yes	Yes
Constant	Yes	Yes	Yes
N	1536	1536	1536

Standard errors in parentheses

* $p < 0.05$, ** $p < 0.01$, *** $p < 0.001$

Other Control Variables include *Age, Female, Trust, Risk Aversion*, and *Game Theory*.

Table S13: Linear combination tests to evaluate between-treatment differences in the likelihood of tied partners to strengthen their ties based on the estimated regression coefficients obtained under all regression specifications shown in Table S12, conditional on the last round action pair being either *CD* or *DC*.

	Specification (1)		Specification (2)		Specification (3)	
Between-treatment Comparison	Difference	P-value	Difference	P-value	Difference	P-value
$S - M$	-0.912	0.036	-1.034	0.002	-1.076	0.001
$MS - M$	-0.003	0.995	-0.001	0.998	-0.049	0.917
$S - MS$	-0.915	0.060	-1.033	0.041	-1.026	0.035

There are no significant between-treatment differences in the likelihood of tie partners to strengthen their ties at 10% level when the last round action pair is either *CC* or *DD*.

Table S14 presents the results of random-effects logit regressions of the likelihood of a pair of participants to mutually cooperate (*CC*). All observations are from rounds 8 to 23. The dependent variable is the pairwise action choice, which equals to 1 if both participants choose to cooperate and 0 otherwise. The first two columns pool all treatments together, while the remaining columns analyze each treatment separately. In all specifications, our baseline for comparison is weak ties. We also include tie strength dummy variables (*Medium, Strong*), and interaction variables between the tie strength dummy variables and the number of mutually cooperative actions achieved in the last three rounds, *Number of CC*. The remaining independent variables used in the regressions are the same as those included in the regressions presented in Table S8.

Table S14: The random-effects logit regressions of the likelihood of mutual cooperation with the standard errors (SEs) clustered at the group (network) level.

**Treatments**	**All (1)**	**All (2)**	**B**	**M**	**S**	**MS**
**Dep. Var: Mutual Cooperation**						
Medium Tie	0.927***	-0.041		-0.139		-0.121
	(0.289)	(0.395)		(0.425)		(1.360)
Strong Tie	1.781***	-0.637			-0.613	-0.442
	(0.330)	(0.430)			(0.560)	(1.155)
(Medium Tie) x Number of CC		0.441		0.510		0.644
		(0.246)		(0.534)		(0.648)
(Strong Tie) x Number of CC		0.874***			0.755***	0.965
		(0.198)			(0.184)	(0.670)
Number of CC		2.501***	2.449***	2.427***	2.786***	2.613***
		(0.126)	(0.143)	(0.606)	(0.332)	(0.614)
Local Clustering (Mean)		0.656	0.704	1.609	-0.879	0.586
		(0.439)	(1.044)	(1.742)	(1.493)	(0.671)
Local Clustering (Diff.)		0.240	0.316	-0.145	-0.774	1.240
		(0.430)	(0.706)	(2.216)	(1.012)	(0.958)
Degree (Mean)		-0.004	-0.049	-0.104	0.129	0.171
		(0.042)	(0.059)	(0.434)	(0.109)	(0.113)
Degree (Diff.)		-0.058	-0.034	-0.019	-0.025	-0.218***
		(0.043)	(0.079)	(0.157)	(0.138)	(0.031)
Frac. of Non-weak Ties (Mean)		-0.862		-0.889	-1.699	-1.914

		(0.442)		(5.169)	(1.593)	(1.093)
Frac. of Non-weak Ties (Diff.)		0.332		-0.125	-1.136	1.449*
		(0.484)		(0.710)	(1.413)	(0.573)
Offer Proposer (Mean)	0.005	-0.005	0.001	-0.005	-0.004	-0.008
	(0.007)	(0.003)	(0.005)	(0.015)	(0.013)	(0.006)
Offer Proposer (Diff.)	-0.001	-0.002	0.002	0.001	-0.013	-0.002
	(0.004)	(0.002)	(0.003)	(0.004)	(0.007)	(0.004)
Round	0.080**	0.005	-0.012	0.032	-0.017	0.041
	(0.026)	(0.018)	(0.020)	(0.027)	(0.101)	(0.027)
Other Control Variables	Yes	Yes	Yes	Yes	Yes	Yes
Constant	Yes	Yes	Yes	Yes	Yes	Yes
N	17216	11413	2743	2670	3559	2441

Standard errors in parentheses

* $p < 0.05$, ** $p < 0.01$, *** $p < 0.001$

Other Control Variables include *Age, Female, Trust, Risk Aversion*, and *Game Theory*.

Table S15: Linear combination tests to evaluate differences in the likelihood of mutual cooperation between different tie strengths based on the estimated regression coefficients obtained under specifications (1) shown in Table S14

	Specification All (1)	
Comparison	Difference	P-value
Medium−Weak	0.927	0.001
Strong−Weak	1.781	0.000
Strong−Medium	0.854	0.039

2.3 Category level

We first describe how elite and peripheral categories are constructed. A participant is categorized as an elite participant if more than $K = 80\%$ of this participant's ties are non-weak ties, otherwise the participant is categorized as a peripheral participant. The rationale for choosing $K = 80\%$ is that a participant's mean and median number of ties is between 5 and 6 across treatments M, S, and MS. Thus, $K = 80\%$ means that a participant with 5 to 6 ties who has at most one weak tie is categorized as being part of the elite. To check whether the choice of threshold K affects our results, we replicate the same analysis using thresholds $K = 100\%$ and $K = 60\%$ in Section 5.3.

We first compare within-treatment differences between elite and peripheral participants. These comparisons are done using Wilcoxon-Mann-Whitney Sign-rank Test (WMW hereafter) for matched samples. Then, for each category we compare it across treatments: we first investigate differences across treatments for elite participants and then apply the same analysis for peripheral participants. As in section 2.1, we run the Kruskal-Wallis test (KW hereafter) [8] to identify treatment effects, and use the Dunn's test (DT hereafter) [9] as our post-hoc test to conduct multiple pairwise comparisons across treatments. We report the results for the last 8 rounds, i.e rounds 15 to 23, and exclude rounds 24 to 25 to remove end-game effects. We start from round 15 to allow enough time for the emergence of elite participants.

We are interested in the following category-level variables:

- *Cooperation*: The number of within-category pairs with mutual cooperation at the end of the round, divided by the total number of possible pairs in a complete within-category network ($n(n-1)/2$ for a category with n nodes).
- *Number of Inlinks*: The total number of existing within-category ties at the end of the round.
- *Size*: The total number of nodes in the category at the end of the round.
- *Proportion of Non-weak Ties*: The proportion of *Number of Inlinks* that are non-weak.
- *Overall Payoff*: The average points obtained by the category members from all interactions at the end of the round.
- *Within Payoff*: The average points obtained by the category members at the end of the round counting only interactions with members of the same category.
- *Inbreeding Homophily Index (IH)*: See Materials and Methods in the main text for a detailed description.
- *Ratio of Within-to-Overall Payoff*: The fraction of the overall payoff of a node that is contributed by within payoff. The ratio is normalized to be between 0 to 1: we set it equal to 1 whenever within payoff is greater than overall payoff, and equal to 0 whenever within payoff is negative.
- *E-P Overall Payoff Difference*: The difference in *Overall Payoff* between elite and peripheral participants. This is only listed in Table S18.
- *E-P Within Payoff Difference*: The difference in *Within Payoff* between elite and peripheral participants. This is only listed in Table S18.

Table S16 documents the average values of the category-level variables of both elite and peripheral participants for rounds 15 to 23.

Table S16: Average values of the category-level variables of both elite and peripheral participants for rounds 15 to 23.

	Elites			Peripherals		
Variable	M	S	MS	M	S	MS
Cooperation	0.84	0.94	0.83	0.54	0.63	0.70
Number of Inlinks	9.26	25.26	17.07	15.31	7.54	16.28
Size	4.84	7.92	4.20	7.62	5.08	8.69
Proportion of Non-weak Ties	0.94	0.95	0.91	0.48	0.50	0.37
Overall Payoff	16.08	64.96	41.63	9.01	32.31	19.28
Within Payoff	12.96	56.29	20.70	1.48	4.99	9.08
Inbreeding Homophily Index	0.17	0.34	0.11	0.16	0.15	0.02
Ratio of Within-to-Overall Payoff	0.47	0.81	0.41	0.28	0.21	0.50

In table S17 we report the results of a Wilcoxon-Mann-Whitney Sign-rank Test (WMW hereafter) for matched sample to test within-treatment differences between elite and peripheral participants for the variables *Cooperation*, *Number of Inlinks*, *Overall Payoff*, and *Within Payoff*. Table S18 and S19 list the p-values obtained from both KW and DT tests conducted on all category level variables for elite and peripheral participants respectively.

Table S17: The results of the Wilcoxon Sign-rank test on within-treatment differences between elite and peripheral participants for rounds 15 to 23. We have $n = 8$ sample size per treatment after aggregation at the group (network) level.

Variables	**Wilcoxon-Mann-Whitney Test** $\binom{z}{P\text{-value}}$		
	M	S	MS
Cooperation	2.380	2.521	2.100
	0.017	0.012	0.036
Number of Inlinks	-1.680	1.960	-1.820
	0.093	0.050	0.069
Overall Payoff	1.820	2.521	2.521
	0.069	0.012	0.012
Within Payoff	2.521	2.521	1.260
	0.012	0.012	0.208

Table S18: The results of the Kruskal-Wallis tests and the Dunn's tests for various category-level variables comparing elite participants across treatments for rounds 15 to 23. We have $n = 8$ sample size per treatment after aggregation at the group (network) level.

**Variables**	**Kruskal-Wallis Tests** $\binom{\chi^2(2)}{P\text{-value}}$	**Dunn's Test** $\binom{z\text{-stat}(col-row)}{P\text{-value}}$		
			M	S
Cooperation	2.122<br>0.346	S	-1.456<br>0.073	
		MS	-0.674<br>0.250	0.732<br>0.232
Number of Inlinks	9.418<br>0.009	S	-2.433<br>0.007	
		MS	0.463<br>0.322	2.813<br>0.002
Size	9.495<br>0.009	S	-2.400<br>0.008	
		MS	0.540<br>0.294	2.858<br>0.002
Proportion of Non-weak Ties	3.560<br>0.169	S	-0.553<br>0.290	
		MS	1.313<br>0.095	1.847<br>0.032
Overall Payoff	8.919<br>0.012	S	-2.985<br>0.001	
		MS	-1.384<br>0.083	1.501<br>0.067
Within Payoff	10.821<br>0.005	S	-3.133<br>0.001	
		MS	-0.636<br>0.262	2.391<br>0.008
Inbreeding Homophily Index	3.896<br>0.143	S	-1.511<br>0.065	
		MS	0.382<br>0.351	1.842<br>0.033

			M	S
Ratio of Within-<br>-to-Overall Payoff	12.055<br>0.002	S	-2.710<br>0.003	
		MS	0.593<br>0.277	3.211<br>0.001
			M	S
E-P Overall<br>Payoff Difference	8.645<br>0.013	S	-2.899<br>0.002	
		MS	-1.025<br>0.153	1.874<br>0.031
			M	S
E-P Within<br>Payoff Difference	11.55<br>0.003	S	-2.687<br>0.004	
		MS	0.460<br>0.323	3.147<br>0.001

Table S19: The results of the Kruskal-Wallis tests and the Dunn's tests for various category-level variables comparing peripheral participants across treatments from rounds 15 to 23. We have $n = 8$ sample size per treatment after aggregation at the group (network) level.

**Variables**	**Kruskal-Wallis Tests** $\left(\frac{\chi^2(2)}{P\text{-value}}\right)$	**Dunn's Test** $\left(\frac{z\text{-stat}(col-row)}{P\text{-value}}\right)$		
			M	S
Cooperation	0.511<br>0.774	S	-0.124<br>0.451	
		MS	-0.672<br>0.250	-0.548<br>0.292
			M	S
Number of Inlinks	6.180<br>0.045	S	1.909<br>0.028	
		MS	-0.424<br>0.336	-2.333<br>0.009
			M	S
Size	8.105<br>0.017	S	1.875<br>0.030	
		MS	-0.920<br>0.179	-2.796<br>0.002

Proportion of Non-Weak Ties	4.515<br>0.105		M	S
		S	-0.424<br>0.336	
		MS	1.591<br>0.056	2.015<br>0.022
Overall Payoff	4.865<br>0.088		M	S
		S	-2.192<br>0.014	
		MS	-1.308<br>0.095	0.884<br>0.188
Within Payoff	3.285<br>0.194		M	S
		S	-0.742<br>0.229	
		MS	-1.803<br>0.036	-1.061<br>0.144
Inbreeding Homophily Index	1.235<br>0.539		M	S
		S	0.707<br>0.239	
		MS	1.096<br>0.136	0.389<br>0.349
Ratio of Within--to-Overall Payoff	6.755<br>0.034		M	S
		S	0.671<br>0.251	
		MS	-1.838<br>0.033	-2.510<br>0.006

Finally, we check whether the *Inbreeding Homophily Index (IH)* for elite participants and peripheral participants are significantly different from zero in order to establish the existence of inbreeding homophily. Table S20 presents the result of Wilcoxon-Mann-Whitney Sign-rank Test comparing the values of *IH* for each category in each treatment with the zero benchmark (no homophily).

Table S20: The results of the Wilcoxon Sign-rank test on *Inbreeding Homophily Index (IH)* for elite and peripheral participants for rounds 15 to 23. We have $n = 8$ sample size per treatment after aggregation at the group (network) level.

Category	**Wilcoxon-Mann-Whitney Test** $\binom{z}{P\text{-value}}$		
	M	S	MS
Elites	1.680	2.100	1.680
	0.093	0.036	0.093
Peripherals	1.820	1.400	0.280
	0.069	0.161	0.779

3 Stability

This section provides the rationale for our choice to focus on rounds 8 to 23 in the analysis. Specifically, in this section we evaluate the stability properties of the network structure. To do so, for each round (except for the first round) we define variable $Change1$ which is the number of new ties formed and the number of old ties removed. For additional robustness, we also define variable $Change2$ which adds onto $Change1$ the number of strengthened ties in each given round. Note that for treatment B, these two stability metrics are the same because it is not possible to strengthen a tie.

We aggregate the two stability metrics by computing the mean value over round intervals $[4,7]$, $[8,11]$, and $[8,23]$ for each treatment. We compare the stability metrics across these round intervals using the Wilcoxon sign-rank test for each treatment. Table S21 shows the values of $Change1$ and $Change2$ in treatments M, S and MS for the above round intervals. In each treatment, the mean value of $Change1$ over the round interval $[8,11]$ is significantly lower ($p < 0.05$) than its value over the round interval $[4,7]$. Similarly, the mean value of $Change1$ over the round interval $[8,23]$ is significantly lower ($p < 0.05$) than its value over the round interval $[4,7]$. The same results holds when we use the $Change2$ metric instead. For this reason, throughout our analysis we focus on rounds $[8,23]$.

Table S21: The stability metrics $Change1$ and $Change2$ in each treatment. The reported p-values are based on the Wilcoxon sign-rank test applied to the comparison between the average value of the metrics over the two indicated time-spans. We have $n = 8$ group (network) level observations per treatment.

Metric	Treatment	Mean Value			P-values	
		[4,7]	[8,11]	[8,23]	[8,11]-[4,7]	[8,23]-[4,7]
$Change1$	1	0.382	0.283	0.216	0.025	0.012
	2	0.322	0.207	0.170	0.017	0.017
	3	0.309	0.193	0.137	0.017	0.012
	4	0.355	0.264	0.196	0.017	0.017
	Pooled	0.342	0.237	0.180	0.001	0.001
$Change2$	2	0.382	0.241	0.199	0.012	0.012
	3	0.379	0.223	0.162	0.012	0.012
	4	0.413	0.302	0.228	0.012	0.012
	Pooled	0.391	0.255	0.196	0.001	0.001

4 Distribution of the proportion of non-weak ties

This section provides the rationale for our choice to focus on two categories of players: elites and peripherals. Specifically, we examine the distribution of participants' non-weak ties to show whether it resembles a bimodal distribution (so that our dichotomous categorization is reasonable).

Figure S2 plots the empirical distribution of the *Proportion of non-weak ties* of each participant in each round, over round 15 to 23. There are 864 observations for each treatment (M, S, MS), hence 2592 observations in total. We exclude treatment B, where all ties are weak by design. Panel A pools the observations across treatments M, S, and MS, while Panel B, C, and D are restricted on observations from specific treatments M, S, and MS respectively. Across all panels, a common feature is that most of the observations are highly concentrated at either the lower end or the upper end, suggesting a pattern of bimodality.

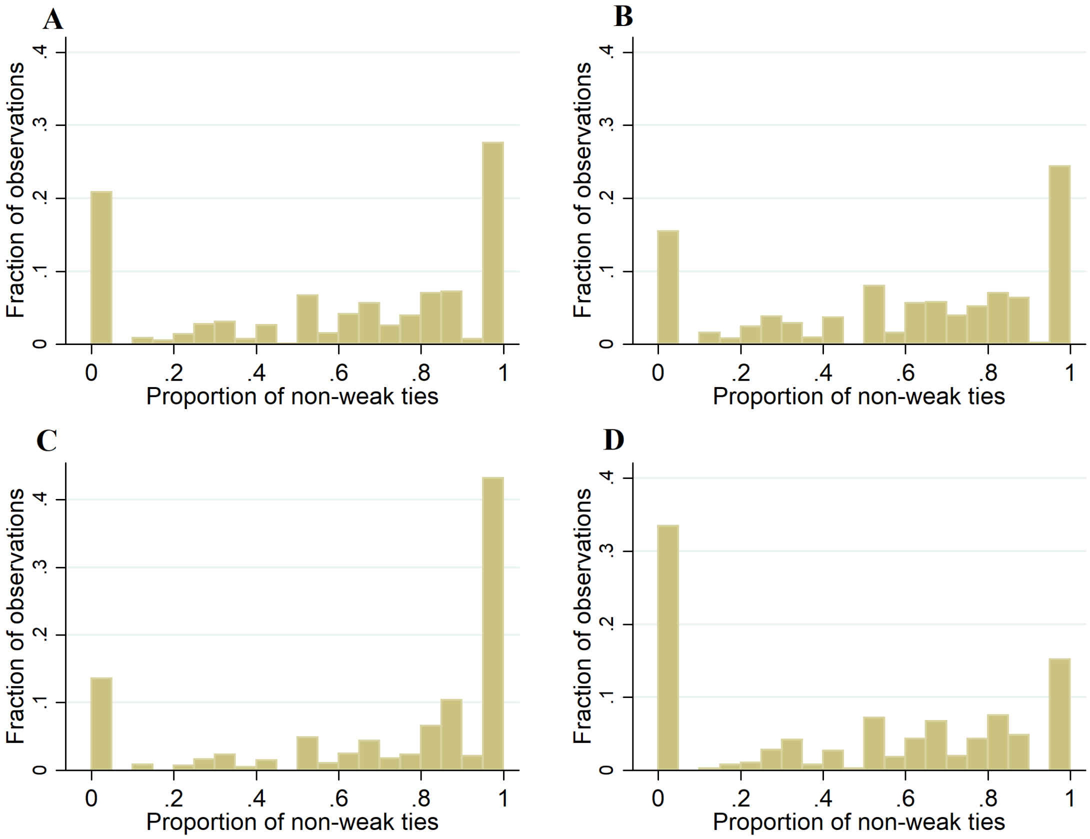

Figure S2: The the empirical distribution of the proportion of non-weak ties. Panel A pools the observations across treatments M, S, and MS. Panel B, C, and D each focuses on treatments M, S, and MS respectively.

To formally test for the bimodality of the distributions, we employ a two-step assessment. We first apply Hartigan Dip Test [10] which test the null hypothesis of unimodality versus the alternative hypothesis of multimodality. A p-value of less than 0.05 indicates the rejection of unimodality. After rejecting unimodality, we then compute the bimodality coefficient (BC) [11] that ranges from 0 to 1. A typical rule of thumb is that a BC value greater than 0.555 indicates bimodality of a given distribution [12]. Table S22 reports the result of this two-step assessment, showing that the distributions of the *Proportion of non-weak ties* are reasonably bimodal.

Table S22: Hartigan dip test and bimodality coefficient for the distributions of *Proportion of non-weak ties* of participants over rounds 15 to 23. We have $n = 864$ sample size per treatment.

Treatment	Hartigan dip test (p-value)	Bimodality coefficient
Treatment M	0.001	0.6355
Treatment S	0.001	0.7976
Treatment MS	0.001	0.6747
All treatments	0.001	0.7038

5 Robustness Check

In this section we present further results to check the robustness of our findings. Section 5.1 replicates the analysis in section 2 using linear regression analysis. Section 5.2 checks the robustness of the statistical analysis in section 2 to the p-value adjustment for multiple comparisons. Section 5.3 replicates the analysis in section 2.3 using alternative thresholds to define the elite category.

5.1 Regression analysis

The analysis in sections 2.1 and 2.3 are based on the non-parametric approach of Kruskal-Wallis test and Dunn's test. In this section, we replicate the analysis using a random-coefficient linear regression model based on panel observations. We find that most of our results remain statistically significant with this alternative approach of analysis.

For the aggregate-level analysis in section 2.1, for each interested aggregate (group)-level variable we specify the linear regression model as follows: for each group i at round t,

$$Variable_{it} = \beta_0 + \delta t + \beta_1 TreatmentM + \beta_2 TreatmentS + \beta_3 TreatmentMS + \alpha_i + u_{it},$$

where α_i is the group-specific random effect and u_{it} the idiosyncratic error term. We have 8 groups for each treatment and we pool observations over round 8 to 23 as in section 2.1. So there are n=512 sample observations in each of the regression. The only exceptions are the regressions for "Cooperation (Non-weak)" and "Proportion of Non-weak Ties (T15-T23)", where the number of observations are respectively n=384 and n=216 . Table S23 below is analogous to Table S4. We report the estimated difference in the treatment coefficients and the associated p-values that are obtained from the linear combination tests. For brevity, we do not report the coefficient for round effect. The definitions of the variables follow from section 2.1.

Table S23: The results of the random-effect linear regression comparing different group (network) level variables for rounds 8 to 23.

**Variables**	**Linear Combination Test** $\binom{\text{coef. diff. between treatments}}{p\text{-value}}$					
	$B-M$	$B-S$	$M-S$	$B-MS$	$M-MS$	$S-MS$
Cooperation	-0.015<br>0.849	-0.155<br>0.048	-0.140<br>0.075	-0.036<br>0.648	-0.021<br>0.790	0.119<br>0.129
Cooperation (Non-weak)			-0.209<br>0.001		-0.065<br>0.314	0.154<br>0.038
Number of Ties	2.367<br>0.526	-2.117<br>0.571	-4.484<br>0.230	4.469<br>0.232	2.102<br>0.574	6.586<br>0.078
Payoff	-5.957<br>0.529	-39.926<br>0.001	-33.969<br>0.001	-16.211<br>0.087	-10.254<br>0.279	23.715<br>0.012
Average Local Clustering	0.037<br>0.433	-0.041<br>0.397	-0.078<br>0.103	0.043<br>0.365	0.006<br>0.903	0.084<br>0.079
Proportion of Non-weak Ties (T15-T23)			-0.116<br>0.183		0.106<br>0.224	0.106<br>0.224
Number of Isolated Nodes	-0.023<br>0.849	-0.023<br>0.849	0.001<br>0.999	-0.102<br>0.409	-0.078<br>0.525	-0.078<br>0.525
Number of Components	-0.016<br>0.905	-0.023<br>0.858	-0.008<br>0.953	-0.086<br>0.512	-0.070<br>0.592	-0.062<br>0.634

Eigenvector Centrality	0.014<br>0.691	-0.025<br>0.463	-0.039<br>0.258	0.053<br>0.126	0.039<br>0.258	0.078<br>0.024

For the category-level analysis in section 2.3, for each interested aggregate (group)-level variable we specify the linear regression model as follows: for each group i at round t,

$$Variable_{it} = \beta_0 + \delta t + \beta_1 TreatmentS + \beta_2 TreatmentMS + \alpha_i + u_{it},$$

where α_i is the group-specific random effect and u_{it} the idiosyncratic error term. Note that Treatment B is excluded by construction. We have 8 groups for each treatment and we pool observations over round 15 to 23 as in section 2.3. There are n=178 sample observations for the regression on elite participants, and $n = 195$ for the regression on peripheral participants. Tables S24 and and S25 below are analogous to Tables S18 and S19. We report the estimated difference in the treatment coefficients and the associated p-values that are calculated from the linear combination tests. For brevity, we do not report the coefficient for round effect. The definition of the variables follow from section 2.3.

Table S24: The results of the random-effect linear regression for various category level variables comparing elite participants across treatments for rounds 15 to 23.

**Variables**	**Linear Combination Test** $\left(\frac{\text{coef. diff. between treatments}}{p\text{-value}}\right)$		
	$M - S$	$M - MS$	$S - MS$
Cooperation	-0.188<br>0.163	-0.092<br>0.513	0.096<br>0.489
Number of Inlinks	-13.853<br>0.013	4.353<br>0.451	18.206<br>0.002
Size	-3.004<br>0.004	0.832<br>0.446	3.836<br>0.001
Proportion of Non-weak Ties	-0.016<br>0.245	0.023<br>0.110	0.039<br>0.006
Overall Payoff	-48.523<br>0.001	-25.347<br>0.061	23.176<br>0.086
Within Payoff	-43.070<br>0.001	-6.896<br>0.524	36.147<br>0.001
Inbreeding Homophily Index	-0.226<br>0.043	0.084<br>0.469	0.310<br>0.007
Ratio of Within- -to-Overall Payoff	-0.307<br>0.003	0.082<br>0.442	0.389<br>0.001
E-P Overall Payoff Difference	-31.662<br>0.003	-4.519<br>0.669	27.143<br>0.010
E-P Within Payoff Difference	-37.804<br>0.001	3.303<br>0.766	41.107<br>0.001

Table S25: The results of the random-effect linear regression for various category level variables comparing peripheral participants across treatments for rounds 15 to 23.

Variables	Linear Combination Test $\binom{\text{coef. diff. between treatments}}{p\text{-value}}$		
	$M-S$	$M-MS$	$S-MS$
Cooperation	-0.065<br>0.143	-0.012<br>0.774	0.052<br>0.236
Number of Inlinks	7.746<br>0.062	-1.057<br>0.797	-8.803<br>0.034
Size	2.514<br>0.029	-1.095<br>0.336	-3.610<br>0.002
Proportion of Non-weak Ties	-0.013<br>0.854	0.107<br>0.114	0.119<br>0.080
Overall Payoff	-22.366<br>0.004	-10.406<br>0.175	11.959<br>0.123
Within Payoff	-3.547<br>0.324	-7.699<br>0.029	-4.152<br>0.247
Inbreeding Homophily Index	0.051<br>0.739	-0.010<br>0.948	-0.061<br>0.691
Ratio of Within--to-Overall Payoff	0.063<br>0.519	-0.218<br>0.020	-0.281<br>0.004

5.2 Multiple comparisons

The analysis in sections 2.1 and 2.3 involve multiple testing across all treatments simultaneously. The presence of multiple comparisons may lead to some spurious significance in the form of incorrect rejection of the null hypothesis. To address this potential problem, we apply the Benjamini-Hochberg adjustment method [13] with a 5% false discovery rate to our post-hoc Dunn's test. After applying the Benjamini-Hochberg adjustment, we find that most of our results remain statistically significant. It is important to note that this adjustment for multiple comparisons is very conservative because we have a low ($n = 8$) number of independent data points given that each independent data point is a network of 12 participants. In other words, the fact that our results are robust to this correction is strong evidence that they are driven by a large effect.

For the aggregate level analysis, Table S26 is the equivalent of Table S4 with the Benjamini-Hochberg adjusted p-values. For the category level analysis, Tables S27 and S28 are the equivalent of Tables S18 and S19 with the Benjamini-Hochberg adjusted p-values.

Table S26: The results of Kruskal-Wallis tests and Dunn's tests with the Benjamini-Hochberg adjustment for multiple comparisons using the data from rounds 8 to 23. We have $n = 8$ sample size per treatment after the aggregation at the group (network) level.

**Variables**	**Dunn's Test** $\binom{z\text{-stat}}{P\text{-value}}$			
Cooperation		B	M	S
	M	0.053<br>0.479		
	S	-1.652<br>0.148	-1.706<br>0.264	
	MS	-0.426<br>0.402	-0.479<br>0.474	1.226<br>0.220
Cooperation (Non-weak)		M	S	
	S	-1.556<br>0.090		
	MS	0.283<br>0.389	1.838<br>0.099	
Number of Ties		B	M	S
	M	0.759<br>0.336		
	S	-0.279<br>0.390	-1.039<br>0.299	
	MS	0.122<br>0.330	0.466<br>0.385	1.506<br>0.396
Payoff		B	M	S
	M	-0.719<br>0.236		
	S	-3.278<br>0.003	-2.558<br>0.016	
	MS	-1.759<br>0.079	-1.039<br>0.179	1.519<br>0.097
Average Local Clustering		B	M	S
	M	1.092<br>0.137		
	S	-0.692<br>0.244	-1.785<br>0.037	
	MS	0.773<br>0.219	-0.319<br>0.375	1.465<br>0.071

Proportion of Non-weak Ties (T15-T23)		M	S	
	S	-1.629<br>0.078		
	MS	0.989<br>0.161	2.616<br>0.013	
Number of Isolated Nodes		B	M	S
	M	-0.986<br>0.972		
	S	-0.027<br>0.489	0.959<br>0.509	
	MS	-0.824<br>0.415	0.162<br>0.523	-0.796<br>0.319
Number of Components		B	M	S
	M	-0.726<br>0.468		
	S	-0.013<br>0.495	0.713<br>0.357	
	MS	-0.767<br>1.000	-0.040<br>0.581	-0.753<br>0.677
Eigenvector Centrality		B	M	S
	M	0.453<br>0.390		
	S	-0.267<br>0.395	0.720<br>0.354	
	MS	1.732<br>0.125	1.279<br>0.201	1.999<br>0.137

Table S27: The results of Dunn's tests with p-values adjusted using the Benjamini-Hochberg adjustment for multiple comparisons. We focus on various category-level variables of elite participants from rounds 15 to 23. After the aggregation at the group (network) level, we have $n = 8$ sample size per treatment after aggregation at the group (network) level.

**Variables**	**Dunn's Test** $\binom{z\text{-stat}}{P\text{-value}}$		
Cooperation		M	S
	S	-1.456<br>0.218	
	MS	-0.674<br>0.250	0.732<br>0.348

		M	S
Number of Inlinks	S	-2.433<br>0.011	
	MS	0.463<br>0.322	2.813<br>0.007
Size	S	-2.400<br>0.012	
	MS	0.540<br>0.294	2.858<br>0.006
Proportion of Non-weak Ties	S	-0.553<br>0.290	
	MS	1.313<br>0.142	1.847<br>0.097
Overall Payoff	S	-2.985<br>0.004	
	MS	-1.384<br>0.083	1.501<br>0.100
Within Payoff	S	-3.133<br>0.003	
	MS	-0.636<br>0.262	2.391<br>0.013
Inbreeding Homophily Index	S	-1.511<br>0.098	
	MS	0.382<br>0.351	1.842<br>0.098
Ratio of Within-to-Overall Payoff	S	-2.710<br>0.005	
	MS	0.593<br>0.277	3.211<br>0.002

		M	S
E-P Overall Payoff Difference	S	-2.899<br>0.006	
	MS	-1.025<br>0.153	1.874<br>0.046
		M	S
E-P Within Payoff Difference	S	-2.899<br>0.005	
	MS	-1.025<br>0.153	1.874<br>0.032

Table S28: The results of Dunn's tests with p-values adjusted using the Benjamini-Hochberg adjustment for multiple comparisons. We focus on various category-level variables of peripheral participants from rounds 15 to 23. After the aggregation at the group (network) level, we have $n = 8$ sample size per treatment after aggregation at the group (network) level.

**Variables**	**Dunn's Test** $\binom{z\text{-stat}}{P\text{-value}}$		
		M	S
Cooperation	S	-0.124<br>0.451	
	MS	-0.672<br>0.752	-0.548<br>0.438
		M	S
Number of Inlinks	S	1.909<br>0.072	
	MS	-0.424<br>0.188	-2.333<br>0.205
		M	S
Size	S	1.875<br>0.046	
	MS	-0.920<br>0.179	-2.796<br>0.008
		M	S
Proportion of Non-Weak Ties	S	-0.424<br>0.336	
	MS	1.591<br>0.084	2.015<br>0.066

		M	S
Overall Payoff	S	-2.192<br>0.043	
	MS	-1.308<br>0.143	0.884<br>0.188
Within Payoff	S	-0.742<br>0.229	
	MS	-1.803<br>0.107	-1.061<br>0.217
Inbreeding Homophily Index	S	0.707<br>0.360	
	MS	1.096<br>0.410	0.389<br>0.349
Ratio of Within- -to-Overall Payoff	S	0.671<br>0.251	
	MS	-1.838<br>0.049	-2.510<br>0.018

5.3 Threshold for the categorization of elite and peripheral participants

In this section we replicate the analysis in 2.3 using two alternative thresholds to define the elite category: $K = 100\%$ and $K = 60\%$. Specifically, four sets of analysis are reproduced: (i) For the cross-treatment comparisons for elite participants, Tables S29 and Tables S30 are the equivalent of Table S18; (ii) For the cross-treatment comparisons for peripheral participants, Tables S31 and Tables S32 are the equivalent of Table S19; (iii) For the within-treatment comparisons between elite and peripheral participants, Tables S33 and S34 are the equivalent of Table S17; (iv) For the significance of *Inbreeding Homophily Index (IH)*, Tables S35 and S36 are the equivalent of Table S20.

Table S29: The results of the Kruskal-Wallis tests and the Dunn's tests for various category-level variables comparing elite participants (threshold $K = 100\%$) across treatments from rounds 15 to 23. We have $n = 8$ sample size per treatment after aggregation at the group (network) level.

**Variables**	**Kruskal-Wallis Tests** $\left(\frac{\chi^2(2)}{P\text{-value}}\right)$	**Dunn's Test** $\left(\frac{z\text{-stat}}{P\text{-value}}\right)$		
Cooperation	0.291<br>0.865		M	S
		S	-0.539<br>0.295	
		MS	-0.250<br>0.401	0.250<br>0.401
Number of Inlinks	9.687<br>0.008		M	S
		S	-2661<br>0.004	
		MS	0.208<br>0.418	2.672<br>0.004
Size	8.679<br>0.013		M	S
		S	-2.487<br>0.006	
		MS	0.256<br>0.399	2.558<br>0.005
Proportion of Non-weak Ties	0.000<br>0.500		M	S
		S	0.000<br>0.500	
		MS	0.000<br>0.500	0.000<br>0.500
Overall Payoff	8.583<br>0.014		M	S
		S	-2.926<br>0.002	
		MS	-1.485<br>0.069	1.224<br>0.111

Within Payoff	7.890<br>0.019		M	S
		S	-1.945<br>0.026	
		MS	0.897<br>0.185	2.698<br>0.003
Inbreeding Homophily Index	0.344<br>0.842		M	S
		S	-0.385<br>0.350	
		MS	0.214<br>0.415	0.570<br>0.284
Ratio of Within--to-Overall Payoff	7.890<br>0.019		M	S
		S	-1.945<br>0.026	
		MS	0.897<br>0.185	2.698<br>0.003
E-P Overall Payoff Difference	4.985<br>0.083		M	S
		S	-2.934<br>0.002	
		MS	-0.990<br>0.161	1.945<br>0.026
E-P Within Payoff Difference	10.385<br>0.006		M	S
		S	-2.298<br>0.011	
		MS	1.450<br>0.074	3.748<br>0.001

Table S30: The results of the Kruskal-Wallis tests and the Dunn's tests for various category-level variables comparing elite participants (threshold $K = 60\%$) across treatments from round 15 to 23. We have $n = 8$ sample size per treatment after aggregation at the group (network) level.

**Variables**	**Kruskal-Wallis Tests** $\binom{\chi^2(2)}{P\text{-value}}$	**Dunn's Test** $\binom{z\text{-stat}}{P\text{-value}}$		
			M	S
Cooperation	1.625<br>0.444	S	-1.237<br>0.108	
		MS	-0.884<br>0.188	0.351<br>0.362

Number of Inlinks	6.236<br>0.044		M	S
		S	-1.379<br>0.084	
		MS	1.114<br>0.133	2.494<br>0.006
Size	4.501<br>0.105		M	S
		S	-1.291<br>0.098	
		MS	0.813<br>0.208	2.104<br>0.018
Proportion of Non-weak Ties	9.245<br>0.009		M	S
		S	-2.157<br>0.016	
		MS	0.778<br>0.218	2.934<br>0.002
Overall Payoff	7.665<br>0.022		M	S
		S	-2.758<br>0.003	
		MS	-1.167<br>0.122	1.591<br>0.056
Within Payoff	7.895<br>0.019		M	S
		S	-2.793<br>0.003	
		MS	-1.131<br>0.129	1.662<br>0.048
Inbreeding Homophily Index	1.295<br>0.523		M	S
		S	-0.672<br>0.251	
		MS	0.460<br>0.323	1.131<br>0.129
Ratio of Within--to-Overall Payoff	3.885<br>0.143		M	S
		S	-1.803<br>0.036	
		MS	-0.212<br>0.416	1.591<br>0.056

			M	S
E-P Overall<br>Payoff Difference	4.835<br>0.089	S	-2.157<br>0.016	
		MS	-0.707<br>0.240	1.450<br>0.074
			M	S
E-P Within<br>Payoff Difference	7.085<br>0.029	S	-2.616<br>0.004	
		MS	-0.884<br>0.188	1.732<br>0.042

Table S31: The results of the Kruskal-Wallis tests and the Dunn's tests for various category-level variables comparing peripheral participants (threshold $K = 100\%$) across treatments from rounds 15 to 23. We have $n = 8$ sample size per treatment after aggregation at the group (network) level.

**Variables**	**Kruskal-Wallis Tests** $\left(\frac{\chi^2(2)}{P\text{-value}}\right)$	**Dunn's Test** $\left(\frac{z\text{-stat}}{P\text{-value}}\right)$		
			M	S
Cooperation	1.635<br>0.442	S	-1.273<br>0.102	
		MS	-0.530<br>0.298	0.742<br>0.229
			M	S
Number of Inlinks	6.204<br>0.045	S	2.033<br>0.021	
		MS	-0.230<br>0.409	-2.263<br>0.012
			M	S
Size	9.264<br>0.001	S	1.998<br>0.023	
		MS	-0.990<br>0.161	-2.988<br>0.001
			M	S
Proportion of<br>Non-Weak Ties	1.635<br>0.442	S	-1.131<br>0.129	
		MS	1.131<br>0.129	2.263<br>0.012

Overall Payoff	6.480<br>0.039	M S<br>S -2.546<br>0.005<br>MS -1.273 1.273<br>0.102 0.102
Within Payoff	3.840<br>0.1466	M S<br>S -1.697<br>0.045<br>MS -1.697 0.000<br>0.045 0.500
Inbreeding Homophily Index	1.085<br>0.581	M S<br>S 1.025<br>0.153<br>MS 0.672 -0.354<br>0.251 0.362
Ratio of Within--to-Overall Payoff	8.720<br>0.013	M S<br>S 0.283<br>0.389<br>MS -2.404 -2.687<br>0.008 0.004

Table S32: The results of the Kruskal-Wallis tests and the Dunn's tests for various category-level variables comparing peripheral participants (threshold $K = 60\%$) across treatments from rounds 15 to 23. We have $n = 8$ sample size per treatment after aggregation at the group (network) level.

**Variables**	**Kruskal-Wallis Tests** $\left(\frac{\chi^2(2)}{P\text{-value}}\right)$	**Dunn's Test** $\left(\frac{z\text{-stat}}{P\text{-value}}\right)$
Cooperation	0.311<br>0.856	M S<br>S -0.218<br>0.414<br>MS 0.361 0.567<br>0.359 0.285
Number of Inlinks	1.847<br>0.397	M S<br>S 0.954<br>0.170<br>MS -0.387 -1.328<br>0.349 0.092

			M	S
Size	3.308<br>0.191	S	1.051<br>0.147	
		MS	-0.793<br>0.214	-1.817<br>0.035
			M	S
Proportion of Non-Weak Ties	5.766<br>0.056	S	0.249<br>0.402	
		MS	2.212<br>0.013	1.887<br>0.030
			M	S
Overall Payoff	3.177<br>0.204	S	-1.740<br>0.041	
		MS	-1.180<br>0.119	0.600<br>0.274
			M	S
Within Payoff	3.385<br>0.184	S	-1.244<br>0.107	
		MS	-1.788<br>0.037	-0.483<br>0.314
			M	S
Inbreeding Homophily Index	2.503<br>0.286	S	0.320<br>0.374	
		MS	1.511<br>0.065	1.140<br>0.127
			M	S
Ratio of Within- -to-Overall Payoff	3.076<br>0.215	S	0.881<br>0.189	
		MS	-0.904<br>0.183	-1.753<br>0.040

Table S33: The results of the Wilcoxon Sign-rank test on within-treatment differences between elite participants and peripheral participants for threshold $K = 100\%$. We perform this for each treatment focusing on rounds 15 to 23. We have $n = 8$ sample size per treatment after aggregation at the group (network) level.

Variables	**Wilcoxon-Mann-Whitney Test** $\binom{z}{P\text{-value}}$		
	M	S	MS
Cooperation	2.380	2.521	1.540
	0.017	0.012	0.123
Number of Inlinks	-2.380	-0.420	-2.521
	0.017	0.674	0.012
Overall Payoff	1.820	2.380	1.540
	0.069	0.017	0.123
Within Payoff	1.2620	2.100	-1.960
	0.208	0.036	0.050

Table S34: The results of the Wilcoxon Sign-rank test on within-treatment differences between elite participants and peripheral participants for threshold $K = 60\%$. We perform this for each treatment focusing on rounds 15 to 23. We have $n = 8$ sample size per treatment after aggregation at the group (network) level.

Variables	**Wilcoxon-Mann-Whitney Test** $\binom{z}{P\text{-value}}$		
	M	S	MS
Cooperation	2.521	2.521	2.380
	0.012	0.012	0.017
Number of Inlinks	1.120	2.380	0.420
	0.263	0.017	0.674
Overall Payoff	2.521	2.380	1.820
	0.012	0.017	0.069
Within Payoff	2.380	2.521	2.380
	0.017	0.012	0.017

Table S35: The results of the Wilcoxon Sign-rank test on *Inbreeding Homophily Index (IH)* for elite participants and peripheral participants given threshold $K = 100\%$. We perform this for each treatment focusing on rounds 15 to 23. We have $n = 8$ sample size per treatment after aggregation at the group (network) level.

Category	**Wilcoxon-Mann-Whitney Test** $\binom{z}{P\text{-value}}$		
	M	S	MS
Elites	0.980	0.980	0.922
	0.327	0.327	0.357
Peripherals	1.820	0.700	1.680
	0.069	0.484	0.093

Table S36: The results of the Wilcoxon Sign-rank test on *Inbreeding Homophily Index (IH)* for elite participants and peripheral participants given threshold $K = 60\%$. We perform this for each treatment focusing on rounds 15 to 23. We have $n = 8$ sample size per treatment after aggregation at the group (network) level.

Category	**Wilcoxon-Mann-Whitney Test** $\binom{z}{P\text{-value}}$		
	M	S	MS
Elites	2.380	2.240	2.521
	0.017	0.025	0.012
Peripherals	1.820	0.431	-0.980
	0.069	0.623	0.327

6 Experimental Instructions (for MS treatment)

Welcome to all of you! You are now taking part in an interactive study on decision making. **Please pay attention to the information provided here and make your decisions carefully. If at any time you have questions to ask, please raise your hand and we will attend to you in private.**

Please note that **unauthorized communication is prohibited**. Failure to adhere to this rule would force us to stop this study and you may be held liable for the cost incurred in this experiment.

Your participation in this study is voluntary. You will receive SGD 2 show-up fee for participating in this study. You may decide to leave the study at any time. Unfortunately, if you withdraw before you complete the study, we can only pay you for the decisions that you have made up to the time of withdrawal, which could be substantially less than you will earn if you complete the entire study.

The amount of your earnings from this study depends on the decisions you and others make. At the end of this session, your earnings will be paid to you privately and in cash. They will be contained in an envelope (indicated with your unique user ID). You will need to sign a claim card given to you and exchange your claim card with your payment.

General Information

Each of you will be given a unique user ID and it will be clearly stated on your computer screen. At the end of the study, you will be asked to fill in your user ID and other information pertaining to your earnings from this study in the claim card. Please fill in the correct user ID to make sure that you will get the correct amount of payment. Rest assured that your anonymity will be preserved throughout the study. You will never be aware of the personal identities of other participants during or after the study. Similarly, other participants will also never be aware of your personal identities during or after the study. You will only be identified by your user ID in our data collection. All information collected will strictly be kept confidential for the sole purpose of this study.

Specific Information

The total duration of this study is approximately 2 hours.

Your total earnings = earnings from Part I + earnings from Part II + earnings from Part III + show up fee (S$2)

All incentives will be denominated in **experimental dollars** (expressed as ECU). The exact conversion rate will be detailed later.

Part I

This part consists of two stages:

Stage 1

In Stage 1, all participants will be assigned as a proposer. As a proposer you will need to propose a division of the **100 ECUs** between you and an unnamed recipient. You and the prospective recipient will receive your proposed division. For example, suppose that in Stage 1 you propose an offer of **y ECUs** for you and **100-y ECUs** for the recipient. Following the proposal, you will receive y ECUs in Stage 1. The recipient will be one of the participants from the **next** experimental session that we will run.

Stage 2

In Stage 2, as a recipient you will receive a division proposed by a **randomly selected proposer from the previous experimental session that we ran** . This means that you will never receive the division proposed by someone who is the recipient of the proposal you made in Stage 1. Thus, you and another participant in this experimental session can never be each other's beneficiary.

Suppose that a participant from previous experimental session proposed an offer of **w ECUs** for himself and **100-w ECUs** for the recipient. If his proposal is selected for you, then you will receive 100 - w in Stage 2.

Earnings in Part I

Your payoff from Part I is the sum of the amount you receive as a proposer (Stage 1) and recipient (Stage 2). Notice that you will only know the outcome of Stage 2 after Part II of the study. In Part I, the conversion rate from experimental dollars (ECU) to Singaporean dollars is:

20 ECU = 1 SGD

Part II

You and 11 other participants will engage in several rounds of interactions. Each round is the same and consists of 3 Stages.

In Stages 1 and 2, you can form and delete links with any of the other participants, which we will explain shortly. If you are linked with another participant then this participant is a ”neighbor” to you.

In Stage 3, you choose either action A or action B, and the choice of action A or B applies to all your neighbors. Action A is color-coded in green and action B is color-coded in blue. The colors are only a visual aid to distinguish the actions and have no meaning. You get points for the action you choose and the action each of the other participants chooses, in the following way:

- You get 0 points if you are not linked with another participant regardless of your choice of action.
- When you are linked with another participant (your neighbor), the number of points you get depends on the actions you and your neighbor choose, and the strength of the link between you and your neighbor. When you first form a link, it has level 1 link strength. The table below illustrates the number of points you get depending on your and your neighbor's actions if you are connected with a link of level l strength:

Level 1 Link Strength

	Neighbor		
You		A	B
	A	3	-5
	B	5	-3

This is what the table says:

- If you choose A and the neighbor chooses A, you get 3 points
- If you choose A and the neighbor chooses B, you get -5 points
- If you choose B and the neighbor chooses A, you get 5 points
- If you choose B and the neighbor chooses B, you get -3 points

You can increase the strength of the link between you and a neighbor if you have been connected with that neighbor for enough rounds. **Specifically, you can choose to increase a level 1 link to a level 2 link if you have been connected with someone for at least 3 consecutive rounds**. The number of points you get if you are connected with a link of level 2 strength is twice of what you get if you are connected with a Level 1 link strength, as illustrated in the table below:

Level 2 Link Strength

	Neighbor		
You		A	B
	A	6	-10
	B	10	-6

Likewise, you can choose to increase a level 2 link to a level 3 link if you have been connected with someone with a level 2 link strength for at least 3 consecutive rounds. The number of points you get if you are connected with a link of level 3 strength is four times of what you get if you are connected with a level 1 link strength, as illustrated in the table below:

Level 3 Link Strength

		Neighbor	
You		A	B
	A	12	-20
	B	20	-12

At the end of each round you will see a summary of the number of points you get with each of the other participant.

You will play 25 rounds of this game for sure. After round 25, there is a 50% chance that the game will terminate in the following round. In other words, in every round after round 25 the experimenters will ”flip a coin” and if the outcome is ”Heads” then the game will terminate.

Stage 1 Link Decisions

In Stage 1, you decide which participants you want to link with. Please consult an example of the screenshot shown in the Attachment (Stage 1) for illustration.

Top of the screen

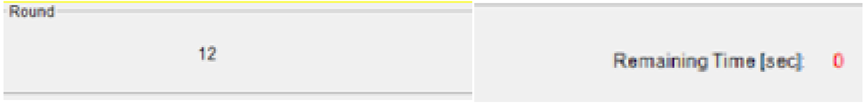

You will be informed of the current round you are in. You also see a countdown timer that indicates the time you have left to make your decisions. In every round, you have 70 seconds to complete Stage 1.

Top-left of the screen

The first row of this table shows the current stage you are in. The figure visualizes your current neighbors.

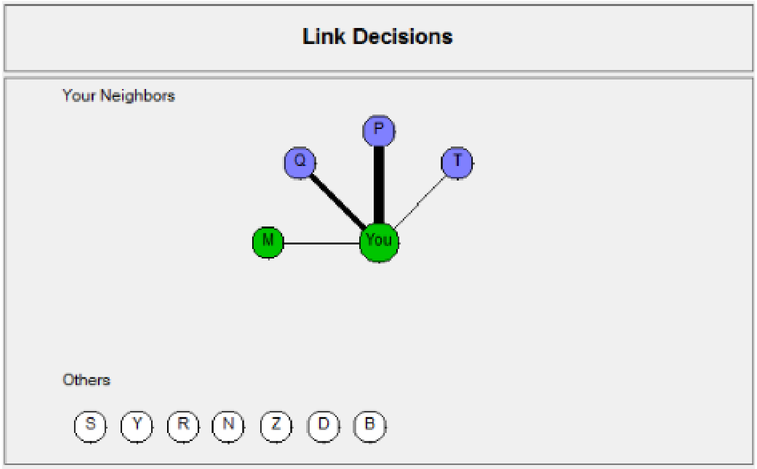

The neighbors you have at the start of each round are the same as the neighbors you had at the end of the previous round. The links means that if you are linked to participant P then participant P is linked to you.

Each circle denotes a participant with an ID. The ”You” circle is always positioned at the center. For example, above you are linked with P, Q, T, and M.

The thickness of the link reflects the link strength. For example, above you have Level 1 link (thin lines) with M and T, Level 2 link (thicker lines) with Q, and a Level 3 link (thickest lines) with P. The color of the circles represents the last action (A or B) that has been chosen by each participant.

Note that no links exist between participants in Round 1, so only the ”You” circle will be displayed.

Center of the screen

This table allows you to make your decisions in Stage 1. The first row of the table (read: Your Last 3 Actions) reminds you of the actions you have chosen in the last 3 rounds. For example, the sequence below means that you chose action A in the last round (the leftmost), action B two rounds ago (the middle slot), and action B three rounds ago (the rightmost).

Your Last 3 Actions: A B B

Neighbor	Past Actions	Link Strength	Unlink	Upgrade
P	B B A	Level 3	☐	
Q	B B B	Level 2	☐	☐
T	B A A	Level 1	☐	☑
M	A A A	Level 1	☐	

In Round 1 you have no links. But from Round 2 onwards, once you have formed links, you can delete a link to any neighbor by ticking the corresponding box under the ”Unlink” column. There is no limit to how many boxes you can tick, and you can also choose not to delete any link by leaving all the boxes unticked.

The table has 5 columns, from left to right:

- **“Neighbor”**: lists the IDs of the participants who are currently linked with you
- **“Past actions”**: lists the last 3 actions chosen by each of your neighbors (leftmost entry: last round action, middle: action chosen two rounds ago, rightmost: action chosen three rounds ago)
- **“Link Strength”**: lists the strength of the link between you and each of your neighbors
- **“Unlink”**: allows you to delete your link to any of your neighbors. For example, ticking the box in the first row will delete your link to participant P.
- **“Upgrade”**: allows you to propose an upgrade of the strength of a link to any of the participants you have been linked to with the same link strength for at least 3 consecutive rounds. For example, you have been linked with T with a level 1 link strength for at least 3 consecutive rounds, and you can propose to upgrade the link strength to level 2 by ticking the box in the third row. Similarly, you can upgrade the link to Q to level 3 by ticking the box in the second row. Notice that you have not been linked to M for the last 3 consecutive rounds, and therefore you cannot upgrade the strength of your link with M in this round.

If you tick both the **“Unlink”** and **“Upgrade”** boxes for the same participant then the following prompt will appear to ask you to confirm your decision and remind you that only the “Unlink” decision will be executed.

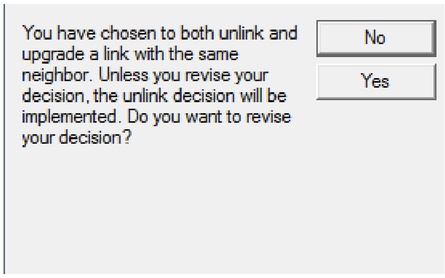

Note that if you delete the link with a participant and then link again with the same participant in a later round, then the link will be of level 1 strength independently of the strength of the link you deleted in the past. The maximum attainable link strength is Level 3.

Right of the screen

This table allows you to make your decisions in Stage 1.

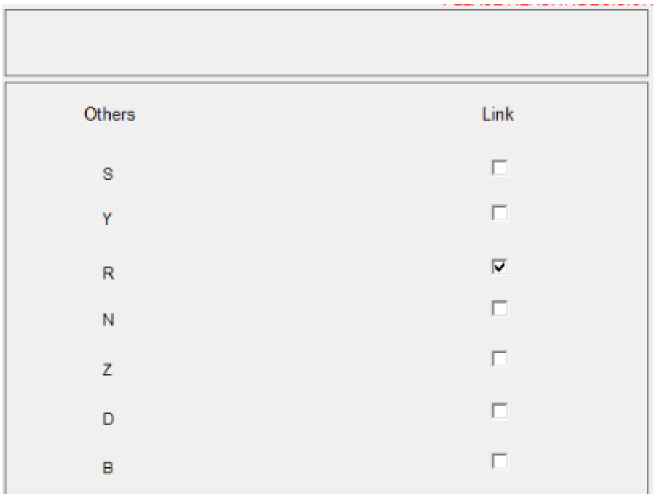

You can propose a link to any other participant by ticking the corresponding box under the ”Link” column. There is no limit to how many boxes you can tick, and you can also choose not to propose any link by leaving all the boxes unticked.

The table has 2 columns, from left to right:

- **“Others”**: lists the IDs of the participants who are not linked with you.
- **“Link”**: allows you to propose a link to any of the participants you are not linked with. For example, ticking the box in the last row will propose a link to participant R.

Stage 2 Yes/No Decision

Please consult an example of the screenshot shown in the Attachment (Stage 2) for illustration. In Stage 2, you decide whether to accept or reject proposals from other participants to add or strengthen a link. You will be shown the following table and you will have to make your decisions in Stage 2.

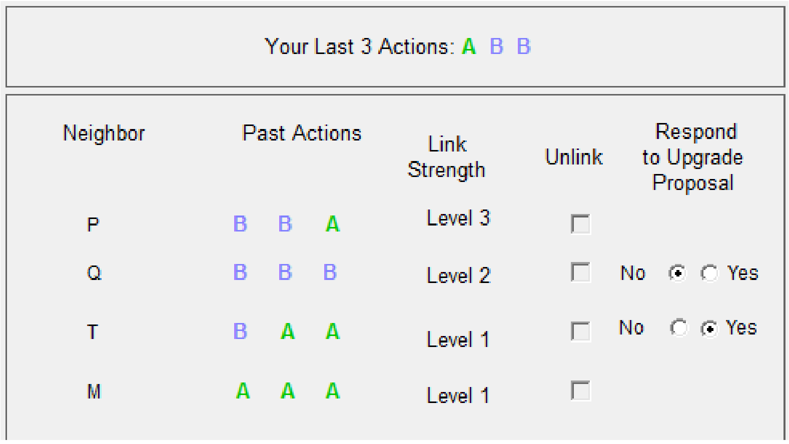

The table is the same as the one you found in Stage 1 except for the last column:

- **“Respond to upgrade proposal”**: allows you to accept or reject a proposal to increase the strength of a link from a neighbor.

For example, look at participants Q and T: under the ”Respond to upgrade proposals” column a “No” and a “Yes” buttons appear. This means that Q and T have sent you a proposal to upgrade the strength of a link. For each proposal, you need to click on ”Yes” to accept it or ”No” to reject it. In the example shown, you have chosen to accept the proposal from T, which means that your level 1 link with T will be upgraded to a level 2 link. You have chosen to reject the proposal from Q, which means your level 2 link with Q will remain a level 2 link.

If there are no buttons then it means that participant has not sent you an upgrade proposal. For example, there are no buttons next to participant M which means that M has not sent you a proposal to upgrade.

Note that if you have sent an upgrade proposal to a participant in Stage 1 and that participant has done the same then the link is automatically upgraded without the need of further approval in Stage 2, and hence no button will be shown.

You will also see the following table.

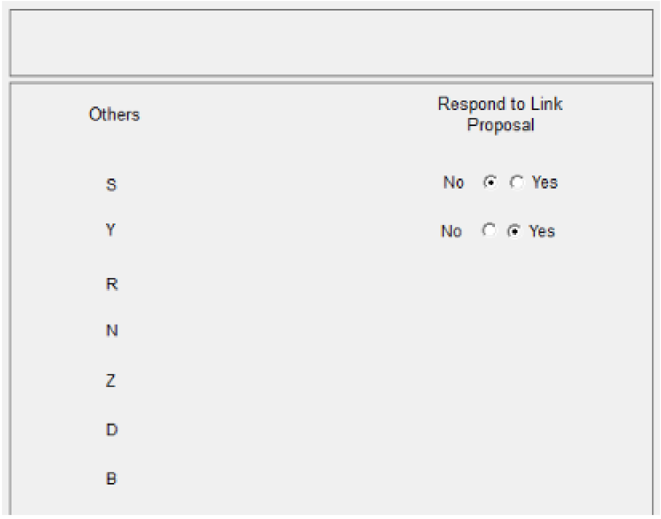

As in Stage 1, the table has 2 columns, from left to right:

- **"Others"**: lists the IDs of the participants who are not linked with you.
- **"Respond to link proposal"**: allows you to accept or reject a link proposal from a participant that is not currently linked with you.

For example, look at participants S and Y: under the "Respond to link proposals" column a "No" and a "Yes" buttons appear. This means that S and Y have sent you a link proposal. For each proposal, you need to click on "Yes" to accept it or "No" to reject it. In the example shown, you have chosen to reject the proposal from S and accept the proposal from Y.

If there are no buttons then it means that participant has not sent you a link proposal. For example, there are no buttons next to participant R which means that R has not sent you a proposal to connect.

Note that if you have sent a link proposal to a participant in Stage 1 and that participant has done the same then the link is automatically formed without the need of further approval in Stage 2, and hence no button will be shown.

Stage 3 Action Choice

In Stage 3, you choose either action A or action B. Please consult an example of the screenshot shown in the Attachment (Stage 3) for illustration. You choose your action in Stage 3 by clicking either the A or B button. You must pick an action in order to proceed.

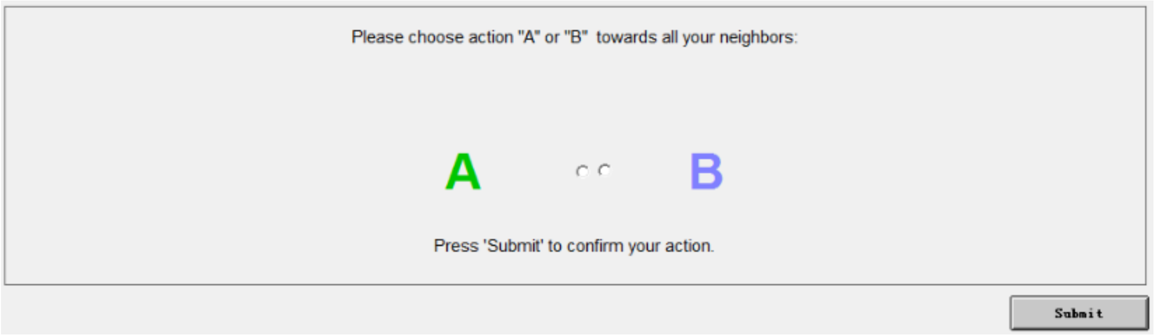

Note that it is possible that you are not linked with any participant (i.e. you have no neighbor) as the result of yours and others' earlier decisions. In such case, you will instead see the message below, and you should click the 'Submit' button to continue.

Action Outcome

After Stage 3, you will see a summary of the points you got with each of the other participants in the round. Please consult an example of the screenshot shown in the Attachment (Action Outcome) for illustration.

You will be shown the following table. The first row of this table reminds you of the action you have chosen in this round. The table shows your neighbors, the strength of your link with each of them, and your neighbors' actions in this round. Note that the table is updated with yours and the others' decisions in the previous Stages.

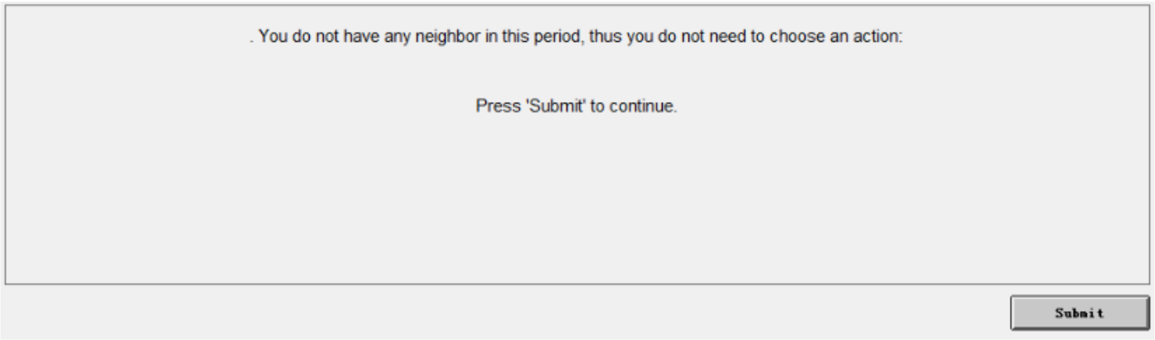

The table has 4 columns, from left to right:

- **"Neighbor"**: lists the IDs of the participants who are currently linked with you.
- **"Action"**: lists the action chosen by each of your neighbors in this round.
- **"Link Strength"**: lists the strength of the link between you and each of your neighbors.
- **"Points"**: lists the points you gained from your and each neighbor's choice of action.

Earnings in Part II

At the end of the Experiment, we will randomly select **6 (six) rounds** for payment. In each of these 6 rounds, we will randomly pick **2 (two) of your potential link pairs** (there are **11** potential link pairs in total in each round). Note that a participant who was not linked with you can be picked, and in that case you will get 0 points for the interaction with that participant. To determine your earnings, we sum the number of points you got with each of the picked participants in each of the 6 rounds. In Part II, the conversion rate from experimental dollars (ECU) to Singaporean dollars is:

3 ECU = 1 SGD for the first 30 ECUs earned

5 ECU = 1 SGD for the subsequent ECUs earned

Part III

In this part of the study you will be asked to make a series of choices. How much you receive will depend partly on chance and partly on the choices you make. The decision problems are not designed to test you. What we want to know is what choices you would make in them. The only right answer is what you really would choose.

For each line in the table you will see in this stage, please indicate whether you prefer option A or option B. There will be a total of 10 lines in the table but just one line will be randomly selected for payment. You do not know which line will be paid when you make your choices. Hence you should pay attention to the choice you make in every line.

After you have completed all your choices, the computer will randomly generate a number, which determines which line is going to be paid out.

Your **earnings for the selected line depend on which option you chose**: If you chose option A in that line, you will receive 20 ECU. If you chose option B in that line, you will receive either 60 ECU or 0.

To determine your earnings in the case you chose option B, there will be a second random draw. The computer will randomly determine if your payoff is 0 or 60, with the chances set by the computer as they are stated in Option B.

Earnings in Part III

In Part III, the conversion rate from experimental dollars (ECU) to Singaporean dollars is:

20 ECU = 1 SGD

Before you collect your payment, we will ask you to answer some questions about yourself while we are preparing your earnings. Please answer truthfully.

Thank you again for your participation! If you have any questions, please raise your hand and an experimenter will come to you.

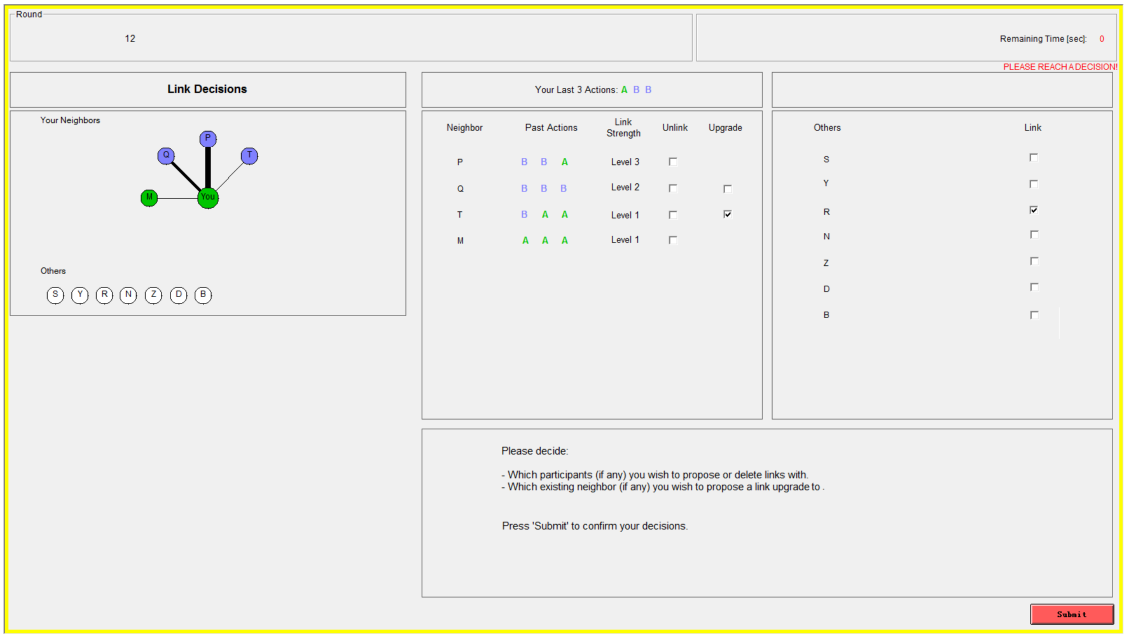

Attachment (Stage 1)

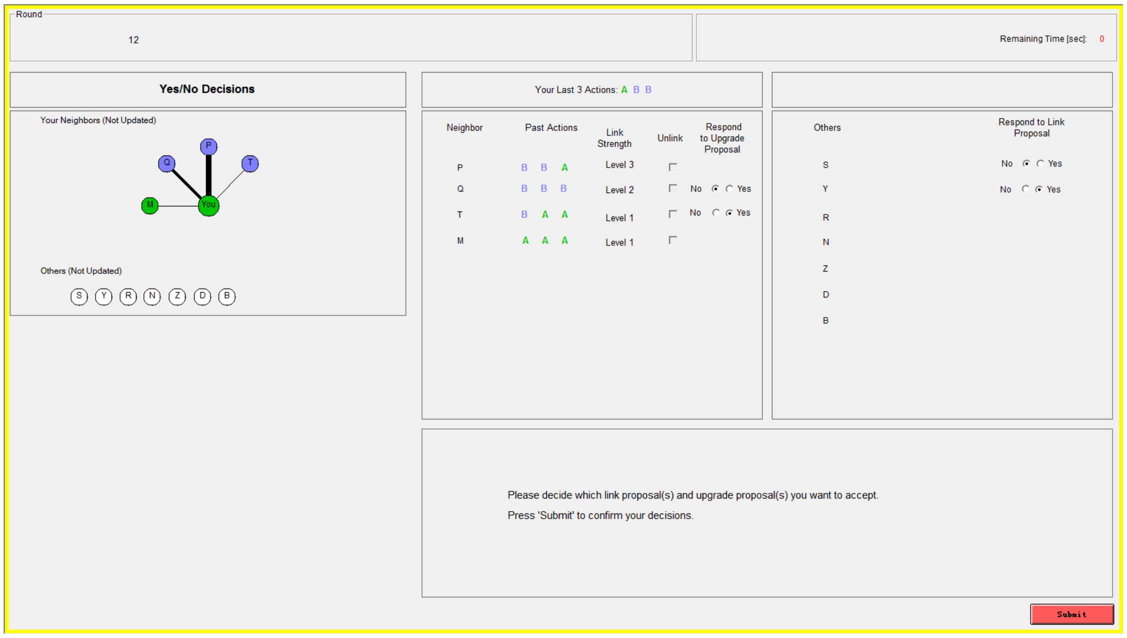

Attachment (Stage 2)

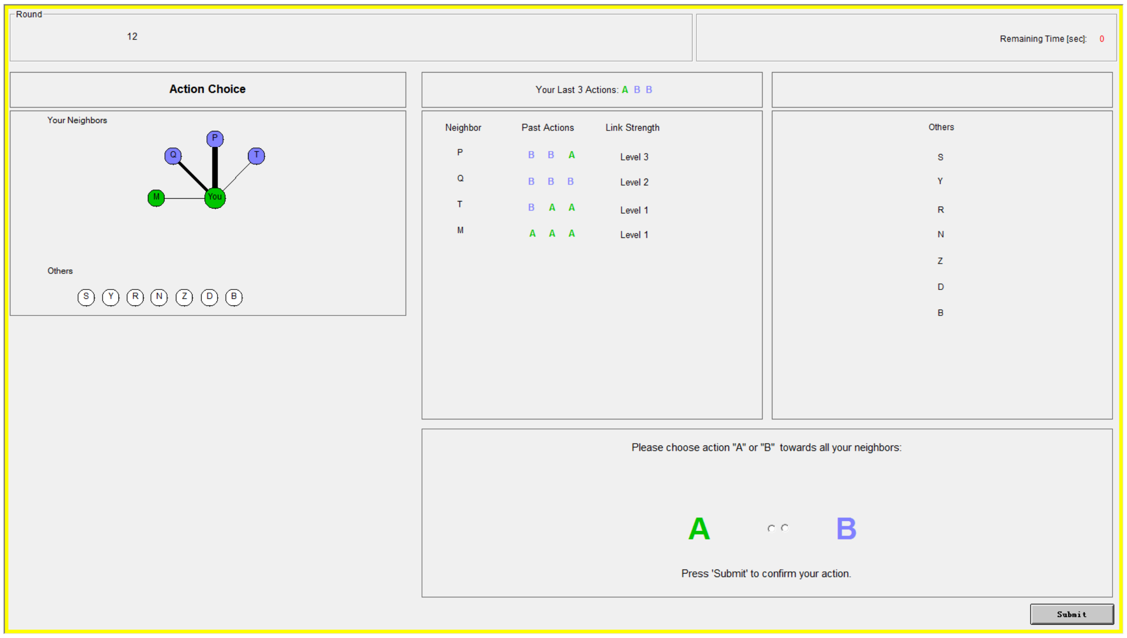

Attachment (Stage 3)

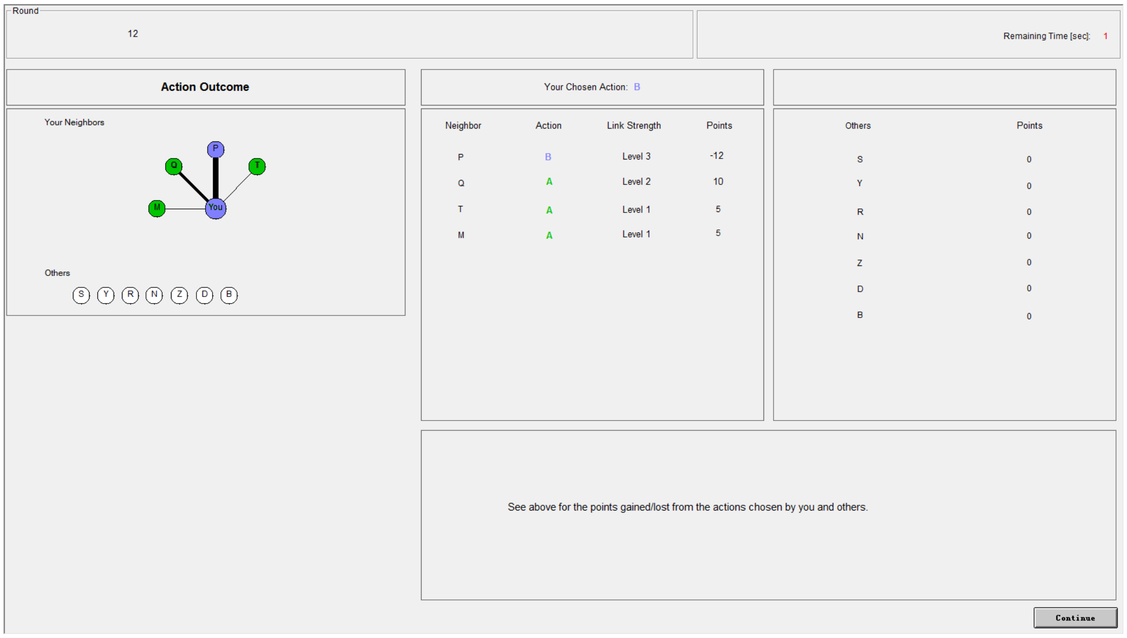

Attachment (Action Outcome)

Supplementary Information References

[1] Fischbacher, U. z-tree: Zurich toolbox for ready-made economic experiments. *Experimental Economics* **10**, 171–178 (2007).

[2] Holt, C. & Laury, S. Risk aversion and incentive effects. *American Economic Review* 1644–1655 (2002).

[3] Rand, D. G., Arbesman, S. & Christakis, N. A. Dynamic social networks promote cooperation in experiments with humans. *Proceedings of the National Academy of Sciences* **108**, 19193–19198 (2011).

[4] Fehl, K., van der Post, D. J. & Semmann, D. Co-evolution of behaviour and social network structure promotes human cooperation. *Ecology Letters* **14**, 546–551 (2011).

[5] Wang, J., Suri, S. & Watts, D. J. Cooperation and assortativity with dynamic partner updating. *Proceedings of the National Academy of Sciences* **109**, 14363–14368 (2012).

[6] Shirado, H., Fu, F., Fowler, J. H. & Christakis, N. A. Quality versus quantity of social ties in experimental cooperative networks. *Nature Communications* **4** (2013).

[7] Gallo, E. & Yan, C. The effects of reputational and social knowledge on cooperation. *Proceedings of the National Academy of Sciences* **112**, 3647–3652 (2015).

[8] Kruskal, W. H. & Wallis, W. A. Use of ranks in one-criterion variance analysis. *Journal of the American statistical Association* **47**, 583–621 (1952).

[9] Dunn, L. C. Multiple comparisons using rank sums. *Technometrics* **64**, 241–252 (1964).

[10] Hartigan, J. A., Hartigan, P. M. *et al.* The dip test of unimodality. *The Annals of Statistics* **13**, 70–84 (1985).

[11] SAS. User's guide, cary, nc, statistical analysis system institute (1989).

[12] Freeman, J. B. & Dale, R. Assessing bimodality to detect the presence of a dual cognitive process. *Behavior Research Methods* **45**, 83–97 (2013).

[13] Benjamini, Y. & Hochberg, Y. Controlling the false discovery rate: a practical and powerful approach to multiple testing. *Journal of the Royal Statistical Society Series B (Methodological)* **57**, 289–300 (1995).
